# Grasping frequent subgraph mining for bioinformatics applications

**DOI:** 10.1186/s13040-018-0181-9

**Published:** 2018-09-03

**Authors:** Aida Mrzic, Pieter Meysman, Wout Bittremieux, Pieter Moris, Boris Cule, Bart Goethals, Kris Laukens

**Affiliations:** 1 Department of Mathematics and Computer Science, University of Antwerp, Antwerp, Belgium; 2 Biomedical Informatics Research Center Antwerp (biomina), University of Antwerp/Antwerp University Hospital, Antwerp, Belgium

**Keywords:** Subgraph mining, Frequent subgraphs, Graph motifs, Biological networks, Pattern discovery, Pattern mining

## Abstract

Searching for interesting common subgraphs in graph data is a well-studied problem in data mining. Subgraph mining techniques focus on the discovery of patterns in graphs that exhibit a specific network structure that is deemed interesting within these data sets. The definition of which subgraphs are interesting and which are not is highly dependent on the application. These techniques have seen numerous applications and are able to tackle a range of biological research questions, spanning from the detection of common substructures in sets of biomolecular compounds, to the discovery of network motifs in large-scale molecular interaction networks. Thus far, information about the bioinformatics application of subgraph mining remains scattered over heterogeneous literature. In this review, we provide an introduction to subgraph mining for life scientists. We give an overview of various subgraph mining algorithms from a bioinformatics perspective and present several of their potential biomedical applications.

**Electronic supplementary material:**

The online version of this article (10.1186/s13040-018-0181-9) contains supplementary material, which is available to authorized users.

## Introduction

Graphs or networks are ubiquitous data types, pervasive in multiple domains, from social sciences to medicine, biology and chemistry. One common problem in graph theory consists of finding the underlying subgraph patterns in graphs, which are also referred to as network motifs or graphlets. In this review, we present a survey of frequent subgraph mining applications that deal with biomolecular graphs, such as interaction networks, protein graph structures and chemical structures.

By definition, a graph consists of nodes and edges. In the molecular biology context, each node corresponds to a biomolecular entity and each edge denotes a certain association or interaction between such entities. For example, a protein structure can be represented as a single graph, in which each node corresponds to an amino acid and each peptide bond is represented as an edge. We can consider any chemical structure as a graph, for example by representing each of its constituent atoms as a node and each molecular bond as an edge. On a cellular scale, the interactions between all proteins can be represented as a graph. In this case each protein is a node, and each physicochemical interaction is represented by an edge.

**Fig. 1:**
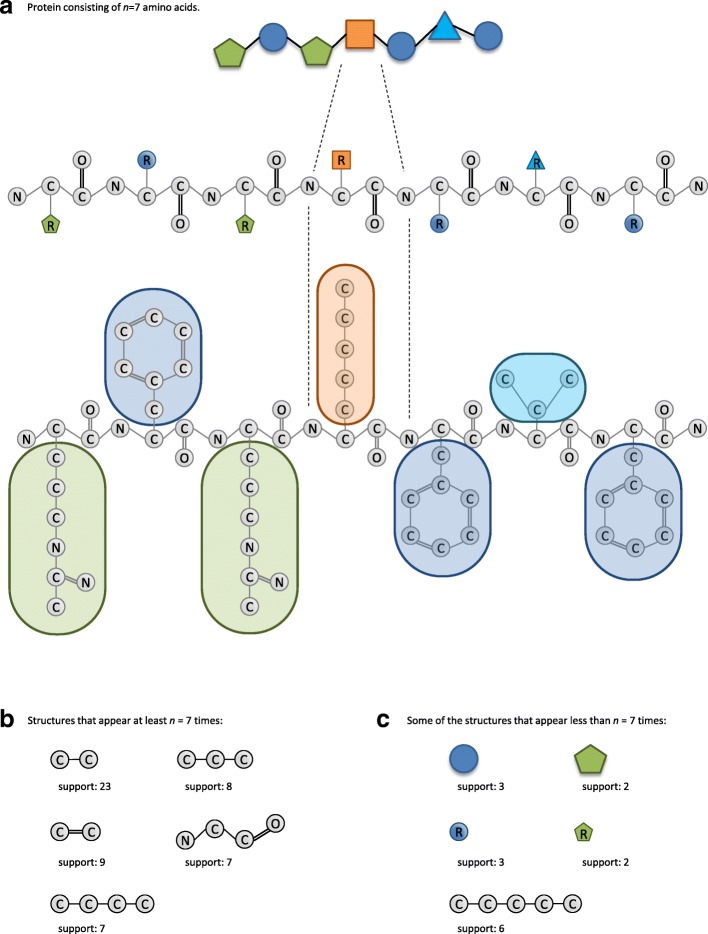
Graph representation of a protein. **a** Protein consisting of *n*=7 amino acids represented as a single graph, visualised at three levels of granularity (bottom: fine granularity, top: coarse granularity). Each node is an atom and each edge is a covalent bond between two atoms. In the first and second representation from the top, the amino acid side chains (R groups) have been replaced with a symbol unique to their content. In the top representation the amino acid backbone has been simplified to a single chain connecting the side groups. **b** Example frequent subgraphs if Support *s*≥*n*, along with the support of each represented subgraph. **c** Example frequent subgraphs if Support *s*<*n*, along with the support of each represented subgraph

Frequent subgraph mining deals with identifying frequently occurring subgraphs in a given graph dataset. A subgraph can be considered frequent if the number of its occurrences within the dataset exceeds a specified threshold *s*. However, how the occurrences of a subgraph are counted depends on the graph setting. For example, subgraph counting will be different for a single graph or for multiple graphs. As an illustration of counting within a single graph, let us represent a protein built up out of *n*=7 amino acids as a single graph, in which each atom is a node and each covalent bond is an edge (Fig. 1a). Figure 1a shows the protein structure at three levels of granularity: (i) as a chain of amino acids; (ii) as a chain of amino acid residues; and (iii) at the atomic level. If we look for subgraphs that appear in the atomic representation graph at least *n* times (so that *s*≥*n*), some very common small two-node subgraphs will be found, such as C-C or C=O, and the protein backbone, i.e. the chemical structure that all amino acids have in common: N-C-C =O, which will occur exactly *n* times (Fig. 1b). Among the subgraphs that occur less than *n* times (*s*<*n*), either individual amino acids will be found or common patterns that occur across amino acids such as C-C-C-C-C (Fig. 1c). It should be noted that the non-overlapping counting scheme was adopted when counting appearances of subgraphs in a large graph, more on which can be found in “In a single graph” section.

Subgraph mining can be applied to various biological data sets and has a wealth of applications, ranging from finding patterns (i.e. network motifs) that explain functional wiring in protein–protein interaction networks to finding shared properties in molecular compounds, relevant for example in the context of drug discovery [1–4]. However, despite its ability to tackle different biological research questions, a straightforward introduction into the bioinformatics applications and context of subgraph mining has thus far been lacking.

With this paper, we aim to cover theoretical concepts and provide a summary of diverse bioinformatics applications of subgraph mining. It must be noted that due to the myriad use cases of subgraph mining this review will not be able to cover the field completely. Instead we provide a general introduction into subgraph mining and its applications without assuming a specific computational background.

## Definitions

In this section, terms are defined that will be used throughout the paper. More detailed definitions are given in Additional file 1. For a more exhaustive introduction into the field of subgraph mining and its algorithms, we refer to [5–7].

### Graphs and subgraphs

A graph *G* is defined as a pair *G*(*V*,*E*) consisting of two sets, a set of nodes *V* and a set of edges *E*⊆*V*×*V*. If the nodes and/or edges of a graph have labels, then such a graph is considered a *labeled* graph. A graph is deemed *directed* if every edge in the graph represents an ordered pair of nodes. If there is no edge orientation in a graph, the graph is deemed *undirected*. Undirected graphs are common for molecular structures as there is typically no specified direction in the chemical bonds. A graph is deemed *connected* if there is a path along the edges that links each pair of nodes; otherwise it is deemed *unconnected*. While most complete chemical structures are connected graphs, many protein interaction networks are for example unconnected. If there is a numeric value assigned to each edge in the graph, then the graph is considered to be *weighted* and the assigned value is called the weight. A graph whose edges have no weight is considered *unweighted*. Weights can be used to denote the certainty of the edge, as estimated by experimental or computational determination. It can also imply the strength of the interaction (for example affinity in interactions between biomolecules).

We say that graph *G*_*s*_ is a *subgraph* of graph *G* if the set of all nodes and the set of all edges of graph *G*_*s*_ are subsets of the set of all nodes and the set of all edges of graph *G*, respectively. A subgraph *G*_*s*_ is an *induced subgraph* of a graph *G* if its set of nodes is a subset of the set of nodes of graph *G*, *V*_*s*_⊆*V*, and its set of edges, *E*_*s*_⊆*E*, consists of all edges that connect nodes in *V*_*s*_ in *G*. In other words, all edges between the selected nodes are preserved. If at least a single edge is different, yet all nodes can be uniquely mapped from the subgraph *G*_*s*_ to the graph *G*, it is a non-induced subgraph.

If there exists a mapping between two graphs such that if two nodes are connected in one graph by an edge, they will be connected in the other graph as well, then such graphs are considered *isomorphic*. In other words, if two graphs are isomorphic, it means that those graphs are considered to be equal. If there are two graphs of different sizes, it is clear they cannot be graph isomorphic, i.e. they cannot be equal. However, if the smaller graph is completely within the larger graph, then the graphs are *subgraph isomorphic*.

### Frequent subgraph mining

In the most straightforward application of subgraph mining, the goal is to find those frequent subgraphs that occur more often than a given threshold. We can distinguish the case of multiple graphs and single graphs when defining how the occurrences of a subgraph are counted, which is sometimes referred to as the *support* of the subgraph. The support will precisely be defined in “Interestingness measures” section. While other definitions of interesting subgraphs do exist, the concept of counting the number of subgraph occurrences is often the first and indeed the most common step in almost all subgraph mining procedures.

## Subgraph mining algorithms

**Fig. 2:**
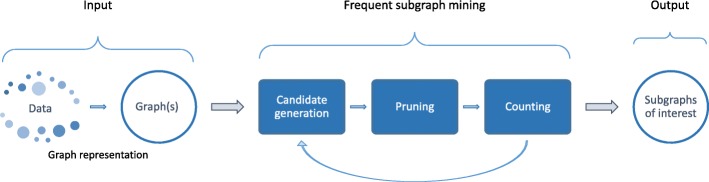
Frequent subgraph mining. Before starting with the mining, input graph data needs to be properly encoded. After that, the first step towards finding frequent subgraphs is generating a set of candidate subgraphs. Then, the frequency of each subgraph in graph dataset will be checked. This is usually preceded by the pruning of the search space and removal of the redundant candidates with the goal of reducing the search space. It is typically an iterative process: larger candidate subgraphs are generated from smaller frequent subgraphs. The counting step outputs the occurrence of the each subgraph that has been checked and this information is used to calculate the subgraph’s interestingness

In this section, we will discuss the algorithmic aspect of subgraph mining. The general procedure of finding subgraphs of interest is shown in Fig. 2.

Before mining for frequent subgraphs the input graph data needs to be properly encoded. Graphs should be represented so that each subgraph has a unique encoding for easier detection of isomorphisms. After the graph(s) have been encoded, the first step towards finding the frequent subgraphs is to generate a set of candidate subgraphs. These candidates are the collection of subgraphs that could be frequent in the graph and need to be checked. After obtaining this set of candidate subgraphs, the next step is to count the number of occurrences of these subgraphs in the graph dataset. Frequency counting is usually the most computationally intensive part of subgraph mining algorithms. For this reason, it is essential to have as few candidate subgraphs as possible. Therefore the counting is commonly preceded by search space pruning and removal of redundant graphs in order to achieve a reduction in the number of candidates. The counting procedure will output how many times each subgraph that has been checked occurs, which can be used to calculate the interestingness of a given subgraph. This can be as straightforward as checking against a fixed threshold. Alternatively, it can include more advanced methods such as calculating statistical enrichment compared to a background distribution.

### Subgraph representation

Graphs and subgraphs can be represented in several ways. The two most commonly used graph representations are the adjacency matrix and the adjacency list.

**Fig. 3:**
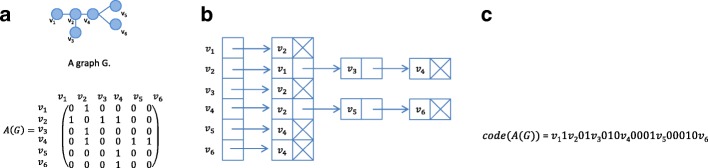
Graph representation. All graph representations refer to the same undirected graph (Graph *G*) in the top left corner but are presented in three different formats. **a** Adjacency matrix of graph *G*. Each element of the graph has a value of one is there exists an edge between the node listed in the beginning of the row and the node listed at the top of the column. Note that this matrix is symmetrical as undirected graphs have no directionality to their edges. **b** Adjacency list of graph *G*. Each entry in the list correspond to a unique node in the graph. A link (represented here by arrows) is then provided to each node to has an edge with the corresponding node. The list of interacting nodes is represented by empty squares pointing to the vertex id. The end of the list of nodes is represented by a square with a cross. **c** Canonical code of graph *G* with adjacency matrix *A*(*G*). This is a string concatenation of the upper triangle of the adjacency matrix representation

**Adjacency matrix** The graph is represented as an *n*×*n* binary matrix; *n* being the total number of nodes in the graph. The position (*i*,*j*) in the matrix can have the value of 0 or 1 for an unweighted graph, depending on whether or not there is an edge between nodes *v*_*i*_ and *v*_*j*_,(*v*_*i*_,*v*_*j*_)∈*E* (Fig. 3a). For weighted graphs, instead of a binary matrix, entries in the adjacency matrix will contain the weights assigned to the corresponding edges.

**Adjacency list** The adjacency list of a graph is an array *A* of length *n*, where *n* is the number of nodes in a graph. Each entry *A*[ *i*] in the array is linked to a list of all nodes connected to the node *v*_*i*_ (Fig. 3b). For directed graphs, each edge (*v*_*i*_,*v*_*j*_) is stored exactly once, while for undirected graphs, each edge (*v*_*i*_,*v*_*j*_) is stored twice; once in a list connected to the *v*_*i*_ node and once in the *v*_*j*_ related list.

**Canonical labeling** Neither the adjacency matrix nor the adjacency list take into account graph isomorphism discovery, which means that it is possible for two graphs which are isomorphic not to share the same adjacency matrix/adjacency list. Canonical labeling ensures that if two graphs are isomorphic they share the same canonical representation. There are several possible ways for canonical labeling. Here, we will present two of the most common approaches.

**Canonical adjacency matrix (CAM)** The CAM is a unique adjacency matrix [8, 9]. Given an undirected graph *G* of size *n* and its adjacency matrix *A*, the CAM code is obtained by concatenating the entries of the upper triangular part of the adjacency matrix *A* in a column-wise fashion. The canonical code of a graph *G* is obtained by comparing all possible codes of the graph and choosing the one with the minimal (maximal) lexicographic value (Fig. 3c).

**Depth-first search (DFS) code** The DFS procedure assigns a unique label to each node while traversing the graph in a depth-first fashion (more about graph traversal strategies in “Subgraph searching and matching” section) [10]. This type of search strategy, specifically the way that subgraphs are extended, results in each subgraph having a unique canonical label.

### De novo subgraph candidate generation

There are several methods for generating subgraph candidates *de novo*, most of which can be possible induced undirected unlabeled candidate generation and extend-based candidate generation.

**Fig. 4:**
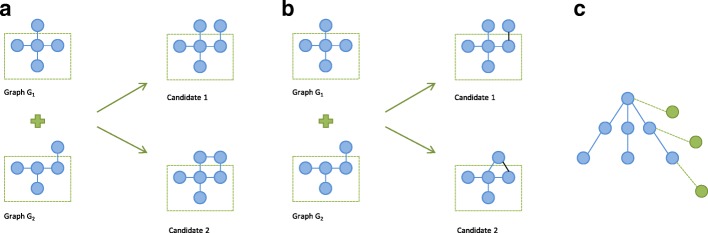
Subgraph candidate generation. **a** Node-based candidate generation, where the join operation will result in a subgraph that is one node larger. **b** Edge-based candidate generation, where the join operation will result in a subgraph that is one edge larger. **c** Right-most path extension, where only edges/nodes that end up in the right-most branch can be added

**Join-based candidate generation** With this method, a new subgraph candidate of size (*k*+1) is created by joining two subgraphs of size *k* that were discovered to be frequent in a previous iteration. Two subgraphs are joined if and only if they share the same (*k*−1)–size subgraph. Depending on the definition of the subgraph size, join-based candidate generation can be either node-based (Fig. 4a) or edge-based (Fig. 4b). Thus k will either be equal to the number of nodes, or the number of edges. The main issue with join-based candidate generation is that multiple candidates can be generated in one join process and equivalent candidates can be generated in a number of different ways, which can lead to the occurrence of duplicate candidates.

**Extend-based candidate generation** A subgraph candidate of size (*k*+1) is generated by extending a frequent *k*-size subgraph with an additional node. The issue with this approach is that there are multiple ways to add the node to a subgraph (it can be added to multiple nodes) which leads to the generation of redundant candidates. One way to solve this is to only add extensions that meet specific criteria. The most common approach in this manner is to use the right-most path extension, i.e. to add the extra node only to a node in the right-most branch (Fig. 4c) [11, 12].

### Subgraph searching and matching

If the goal of subgraph mining is to find all subgraphs that occur more than a set number of times, the subgraph candidates can be pruned based on the Apriori principle. This states that the number of occurrences of a subgraph can never be higher than those of the subgraphs that it contains. Therefore if a subgraph does not pass the frequency threshold, any future candidate graph containing this subgraph need no longer be checked. Two types of search strategies exist that are based on this principle: breadth-first search (BFS) and depth-first search (DFS).

**Fig. 5:**
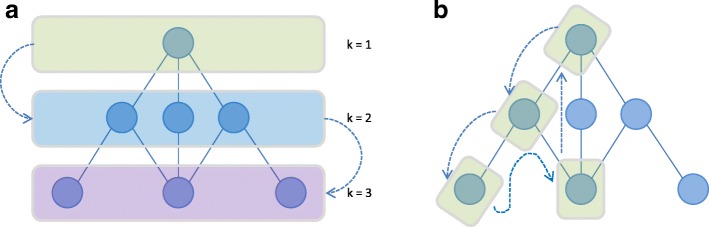
Search strategy. **a** Breadth-first search (BFS), which will exhaust all relevant candidate subgraphs of a given size before proceeding to the next size. Candidate subgraphs of a larger size are then generated based on joining two subgraphs of a smaller size. **b** Depth-first search (DFS), which will explore an entire branch of the subgraph lattice before restarting at the top with a different branch. New candidate subgraphs are extended following a fixed set of rules until it drops below the frequency threshold or has reached a predefined maximum size

**The breadth-first search (BFS) strategy** The BFS strategy checks the support of all candidates of a certain size, before moving to the next level; i.e. first all possible candidate subgraphs of size *k* will be generated and checked for support, subsequently the frequent subgraphs will be retained and used to generate the candidate subgraphs of size (*k*+1). A BFS is necessary if the subgraph candidates are generated by the join-based generation method. For example, to generate the (*k*+1)-size subgraph candidates two *k*-size frequent graphs are needed, which means that all frequent subgraphs of size *k* need to be determined first (Fig. 5a). This approach accounts for effective candidate pruning, but at the cost of a high memory usage.

**The depth-first search (DFS) strategy** The DFS strategy first checks the support of a candidate subgraph of size *k*; if this subgraph is frequent it will be extended to size (*k*+1) and checked for support again (Fig. 5b). The subgraph will continue to be extended until it is no longer frequent. Compared to the BFS this approach requires less memory but at the cost of less effective pruning.

**Fig. 6:**
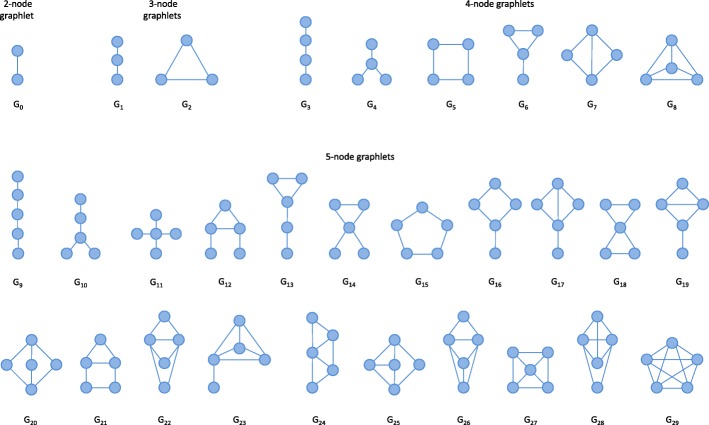
Graphlets. All 30 possible graphlets of node size 2–5. These are undirected subgraphs featuring every valid combination of edges between a given number of nodes

There is another generation and search strategy that is common in bioinformatic approaches, where all possible subgraphs are generated in advance and each is subsequently tested in turn. This is only feasible if the candidate space is fairly small so that there are few possibilities, for example only unlabeled subgraphs of a fixed small size, or if there is prior knowledge about what kind of subgraphs need to be tested, such as feed-forward loop structures. The most common of these deal with induced subgraphs called *graphlets*, which are very common for the analysis of interaction networks [13–15]. Graphlets are all possible induced undirected unlabeled connected subgraphs up to a given size, usually 5 nodes so that there are 30 unique graphlets (Fig. 6). In these cases, all subgraphs that need to be checked can be predefined. Therefore, no pruning is needed and the occurrence of every defined subgraph is counted.

### Interestingness measures

After obtaining a set of candidate subgraphs, the frequency of these subgraphs needs to be counted. In most cases, frequency counting is the most computationally intensive part of the subgraph mining algorithms.

**Frequent subgraph mining.** Let *D* be a graph dataset (a graph database or a single graph) and let *σ* be a user-defined minimal support threshold. Frequent subgraph mining deals with finding all subgraphs *G*_*S*_ in *D* such that:

1 $$\begin{array}{@{}rcl@{}} \text{support}\left(G_{S},D\right) \geq \sigma \end{array} $$

#### In multiple graphs

**Fig. 7:**
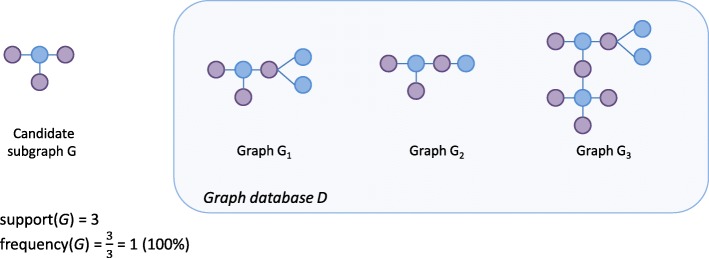
Support count in multiple graphs. The candidate subgraph *g* occurs in all three of the graphs with the graph database *G* and thus has a support of 3. Even though it occurs multiple times in graph *G*_3_, it is only contained once for this graph

When searching across multiple graphs, the count of a subgraph is usually defined as the number of graphs that contain it, independent of the number of times that the subgraph actually occurs within each of these graphs (Fig. 7).

**Support of a subgraph (in multiple graphs)** Let *D* be a graph database. The support of a subgraph *G*_*S*_ is defined as the number of graphs in *D* that contain *G*_*S*_ as a subgraph:

2 $$\begin{array}{@{}rcl@{}} \text{support}\left(G_{S},D\right) = |\left\{G \mid G \in D,G_{S} \subseteq G \right\}| \end{array} $$

**Frequency of a subgraph (in multiple graphs)** Given a database of graphs *D* the frequency of a subgraph *G*_*S*_ is defined as the fraction of all graphs in *D* that contain subgraph *G*_*S*_:

3 $$\begin{array}{@{}rcl@{}} \text{frequency}(G_{S},D) = \frac{\text{support}(G_{S},D)}{|D|} \end{array} $$

**Statistical significance**

The most frequent subgraphs are not necessarily the most relevant for a given research problem. The statistical significance of a subgraph is usually determined by checking how often that subgraph appears in a background graph database, for example one where the edges have been randomized within each graph. Statistical significance can then be used as a post-processing step to filter out insignificant frequent subgraphs or to mine for subgraphs that are significantly associated with a certain subset of the database (i.e. subgroup discovery) [16–18] (Fig. 9).

#### In a single graph

**Fig. 8:**
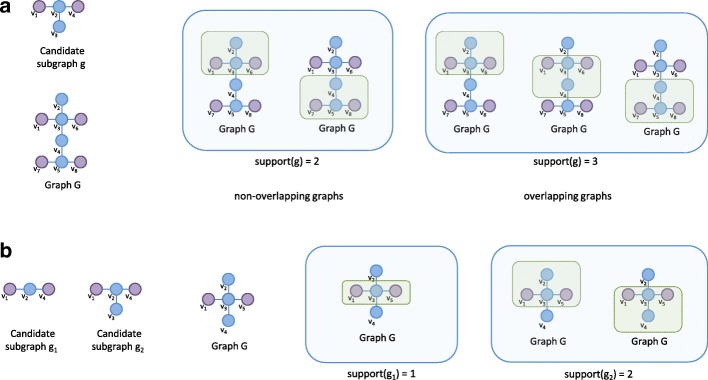
Support count of candidate graph *g* in a single graph *G*. **a** Non-overlapping versus overlapping graphs. For non-overlapping counts, only those instances of the subgraph are counted that are entirely unique and thus don’t share any of its nodes or edges with another instance. Overlapping counts allow subgraphs to share nodes and edges, as long as there is at least one node/edge difference with another subgraph (to distinguish between identical instances). **b** Non-monotonic example. Subgraph *g*_1_ has a support of 1, despite that the larger subgraph *g*_2_ that contains it has a support of 2. Subgraph *g*_1_ will only be counted once in graph G as no other instances is available that uses a different set of nodes or edges. This general procedure is equivalent for both multi- and single graph problems

**Support of a subgraph (in a single graph)** Given a graph *G* the support of a subgraph *G*_*S*_ is defined as the measure of occurrence of subgraph *G*_*S*_ within graph *G*. For a single graph, the support and frequency are the same. However, counting the support within a single graph is not as straightforward as for multiple graphs [19, 20]. First of all, different occurrences of a subgraph can overlap and this can lead to several difficulties. When dealing with overlapping subgraphs there are two approaches to count the occurrences of a subgraph within a single graph: the first approach takes into account only non-overlapping subgraphs; the second approach allows an overlap between various subgraphs (Fig. 8a). In the first approach, only those subgraphs which share no edges (or no nodes) are considered non-identical, while in the second approach two subgraphs are deemed non-identical if they differ by at least one edge (or one node).

**Fig. 9:**
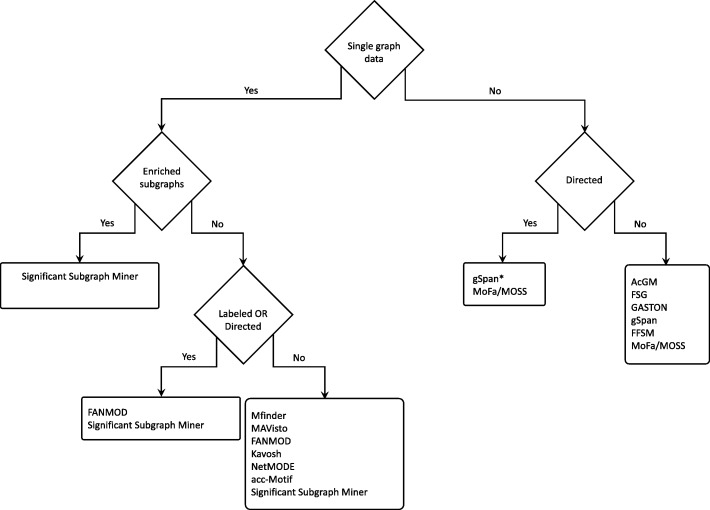
Overview of statistical significance testing for subgraphs. The significance of a list of frequent subgraphs, e.g. *G*, found in the graph database *D* can be tested by comparing to a background database *B*. This database is often established by randomly permuting either the labels or the edges of *D* to represent a relevant background set. This permutation procedure is performed a large number of times (1000s) and thus generates a large set of random graphs. The support of *G* is then enumerated within *B*, which establishes the distribution of *G* at random, which can be presented with a density graph as seen here. The statistical significance can then be reported with a P-value, which corresponds to the chance of seeing a support value that is as high or higher than the observed support in *D* for the random graphs collected in *B*. If this P-value falls beneath a predetermined significance cut-off (often 0.05 with a correction for multiple testing), the subgraph is reported as significant

When considering how to deal with overlapping subgraphs within a single graph, one must also take into account the Apriori property. This states that the count of the larger set (graph) cannot be greater than the count of any of its subsets (subgraphs), and allows pruning of a large portion of the search space. However, for overlapping subgraphs simply counting the number of occurrences of a subgraph within a graph might not adhere to this property because parts of overlapping subgraphs are shared. This is shown in the example in Fig. 8b. If *g*_1_ is a subgraph of *g*_2_, it should have an equal or higher count. However, it is clear that *g*_2_ has a higher count. To this end, other overlap support measures have been proposed, a prominent one being the size of the maximum independent set (MIS) measure of graphs [21–23].

**Statistical significance** Sometimes the subgraphs of interest are not the ones that occur most frequently, but the ones that are significantly enriched in certain nodes. The significance of a subgraph can be determined based on a statistical background distribution (Fig. 9). Significant subgraphs can then be those that occur more frequently than expected in a random graph [24].

**Sampling** Sometimes the graph is simply too large to be efficiently analyzed and thus the use of sampling approaches is required. Graph sampling considers only a representative sample of the graph instead of the entire graph. This means that when searching for frequent subgraphs, instead of enumerating the entire graph, only a representative sample is taken into account. More on sampling in large graphs can be found in [25–27].

## Biomedical subgraph mining

**Fig. 10:**
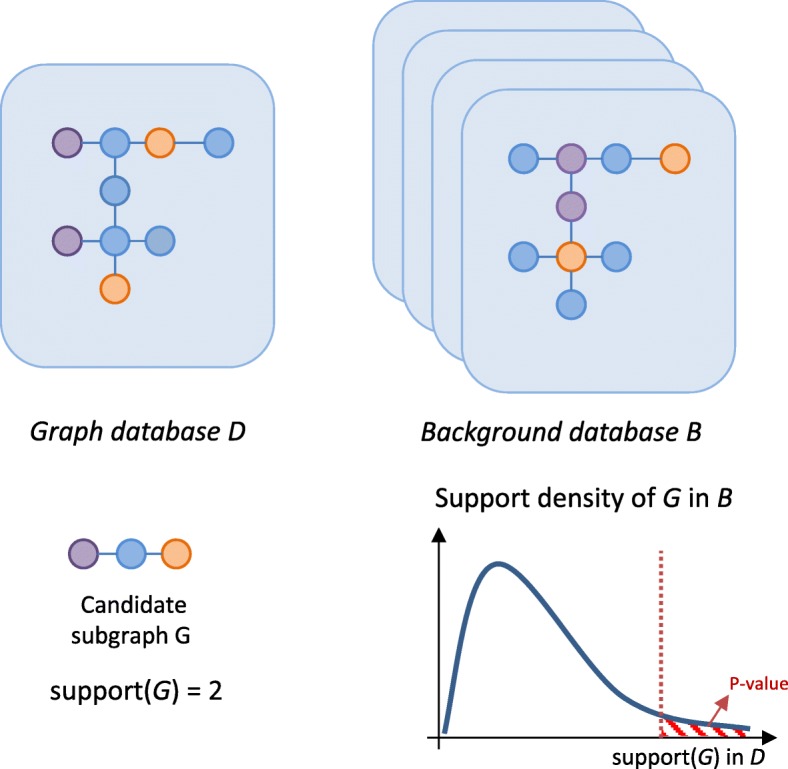
Flowchart with the various subgraph mining tools available for different problems. When choosing the right subgraph mining tool for a specific problem, certain considerations must be made. The biggest distinction is if the dataset is or can be represented as a single graph or as multiple graphs. Further choices can then be made specific for each of these settings. Several tools have the same use cases and will return identical outcomes, so that the major distinction is on platform support, ease of use and speed/memory requirements. *Note that gSpan can be made to work with directed graphs with minor modifications [5]

Subgraph mining methods have been applied to a wide variety of biomedical problems. The reason for this is that many biological features and datasets can be represented as a graph or a collection of graphs, which enables usage of these methods. Often this requires conversion from the typical format into a graph representation, and in some cases this incurs a loss of information. However, in these cases, the advantage of being able to use subgraph mining approaches far outweighs any disadvantages. A broad distinction can be made between the problems that deal with multiple graphs, and those that deal with single graphs. The following is a non-exhaustive description of some of the most common usages of subgraph mining in bioinformatics, together with frequent subgraph mining algorithms and available implementations. A flowchart detailing the differences in various subgraph mining implementations, as featured here, can be found in Fig. 10.

### In multiple graphs

#### Application in bioinformatics

As introduced in “Interestingness measures” section, the goal of subgraph mining in multiple graphs typically concerns finding those subgraphs that occur at least once in a sufficiently large number of these graphs. As such, these applications focus on finding common subgraph patterns that are ubiquitous in the graph collection, which may indicate their biological relevance.

**Molecular data**

One of the archetypical examples is subgraph mining of a database of (bio-)molecules for common subgraph patterns. Indeed such datasets are often used as benchmarks for the development of novel subgraph mining approaches [10, 28–31]. In this instance, each graph in the collection represents a single molecule. The atoms of the molecule are then represented as nodes, and the covalent bonds between the atoms as edges. The edges here are therefore undirected, however they are sometimes labeled to denote the type of bond, or weighted to denote the strength or length of the bond. The goal of the subgraph mining often involves the discovery of common molecular structures, such as benzene rings or disulfide bonds that can be related to a chemical, physical or biological feature. This is for example useful for the discovery of new drug compounds. By mining those molecules that are known to have a specific drug activity for common patterns, one can identify potential new candidates by searching for unstudied molecules that contain the identified subgraphs [32–34].

**Protein structures**

The three-dimensional structure of a protein is often represented as a graph for mining purposes. In these cases, this will usually not be at the level of individual atoms, as described for other biomolecules in the previous section, but at the level of amino acid residues. The graph representation will then typically not concern itself with the chemical bonds that exist to build up the protein structure, but will describe those amino acids that are in close proximity, regardless of whether or not an actual bond exists between them. The nodes of the graph thus represent the different amino acid residues of the protein, and two nodes are connected with an edge if the residues are in close spatial proximity in the three-dimensional protein structure. Common criteria that define *close proximity* include a fixed maximum distance, often 7 or up to 11.5 angstrom, between the *α*-carbon atom of the amino acids, i.e. the carbon atom that the residue chain is attached to, or another fixed position in the amino acid structure [4, 35, 36]. These distances are defined such that it can be supposed that if two amino acids are sufficiently close they likely interact or can influence each other in some manner. The nodes are then labeled with the type of amino acid, e.g. alanine, leucine and so on. The edges are undirected and usually left unweighted. The mined subgraphs therefore consist of patterns of amino acid residues that occur with high frequency in the collection of proteins. While the goal of this mining can be fairly diverse, it most commonly involves correlating the found subgraphs with protein functions. Indeed, these patterns often represent evolutionary conserved three-dimensional structures or domains, which may indicate that they have a biological function in these proteins [35, 37], and can in turn be used as features for functional prediction.

**Phylogenetic trees** The construction of phylogenetic trees that represent similarity between sequences is a common staple in bioinformatics. As a tree is a specific type of graph, many of the approaches detailed here can be readily applied to collections of trees to find so-called subtrees. In this case, the nodes represent the different genes, proteins, species or other biological entities of interest that were contained in the original dataset for comparison, supplemented with the branch nodes signifying inferred ancestors. In the case of a rooted tree, one branch node will be labeled as the *root node*. The edges denote the relationship between the studied entities and their ancestors. These edges can be weighted with the branch length, however these are often not used in the mining process. The actual mining proceeds in a manner very similar to that of normal subgraph mining as already outlined in this review. However, trees do have specific properties that can be utilized to speed up the mining process. The mining of phylogenetic subtrees is most relevant in the context of the discovery of robust phylogenetic relationships in a larger collection of phylogenetic trees. For example, many methods used to study the evolutionary relationship between sequences generate a large number of different trees, each representing a potential phylogenetic possibility. A common approach is therefore to search for the consensus tree, i.e. the single tree that contains those phylogenetic relationships that most of the generated trees agree on. By mining the different solutions, those frequent subtrees can be found, and combined into the consensus solution [38–40].

#### Subgraph mining tools available for bioinformatics use

Here we present a short overview of the algorithms and available implementations to mine subgraphs in multiple graph settings. Table 1 summarizes the information on the algorithms and tools for multiple graphs. A more detailed overview of these frequent subgraph mining algorithms and their implementations can be found in [41].

**Table 1:** Overview of subgraph mining algorithms and tools for the multiple graph setting

Algorithm	Interface	Programming language	Website
AcGM [9]	Command line	- (Binary executable)	[76] ^a^
FSG [43, 44]	Command line	- (Binary executable)	[77]
GASTON [30]	GUI	Java	[78] ^a^
gSpan [10]	GUI	Java	[78] ^a^
FFSM [29]	GUI	Java	[78] ^a^
MoFa/MOSS [34]	GUI	Java	[79]

^a^These tools are no longer supported by the authors, and we cannot guarantee the reliability of the supplied URLs; we supply them for convenience

**AGM/AcGM** The Apriori graph mining algorithm (AGM) [9] represents graphs by an adjacency matrix and searches for frequent subgraphs in a BFS manner. Candidate subgraphs are created by join-based candidate generation, which in this case is node-based (i.e. in each iteration one node is added to the subgraph). AcGM [42] is a version of AGM that only looks for frequent connected subgraphs. The AcGM executable for Linux is available.

**FSG** FSG [43, 44] represents graphs with adjacency lists and uses a BFS strategy to find frequent subgraphs. As opposed to AGM (and AcGM), FSG generates candidate subgraphs by adding a new edge in each iteration, i.e. it uses edge-based candidate generation. An FSG implementation can be found as a part of the PAFI software, which contains executables for Linux (PAFI is written in ANSI C and C++).

**GASTON** The Graph/Sequence/Tree extraction (GASTON) [30] algorithm divides the subgraph mining procedure into different subtasks by evaluating subgraphs from a lower to a higher complexity. It starts by looking for paths first, continues with free trees and in the final stage it looks for cyclic graphs. Here, a path is a sequence of nodes connected by edges, which includes every node only once; a free tree is a connected graph which does not contain any cycle; and a cyclic graph is a graph which contains at least one cycle. The source code for the Gaston algoritm is available in C++ and Java (Parallel and Sequential Mining Suite (ParSeMiS), ParMol package).

**gSpan** gSpan [10] is one of the most well known algorithms for frequent subgraph mining. This algorithm traverses the search space in a DFS manner and creates new subgraphs using the right-most path extension with minimum DFS code. This way it combines the creation of new subgraph candidates with subgraph isomorphism testing. The source codes of gSpan is available in C++ (gBoost toolbox) and Java (Parallel and Sequential Mining Suite (ParSeMiS), ParMol package).

**FFSM** The Fast Frequent Subgraph Mining (FFSM) [29] algorithm uses a CAM representation of graphs and traverses the search space in a DFS manner. It stores an embedding set for each discovered frequent subgraph and thus avoids subgraph isomorphism testing. Both binaries and Java source code (part of ParMol package) for FFSM are available.

**MoFa** The Molecular Substructure Miner (MoSS)/Molecular Fragment Miner (MoFa) [34] is an algorithm inspired by eclat [45], designed for finding frequent fragments (frequent connected subgraphs) in molecular data. It uses adjacency matrices for graph representation and traverses the search space in a DFS manner. It is implemented as part of the ParMol package and in the Molecular Substructure Miner (MoSS). Although it was initially designed for molecular data, MoSS can also work with other types of graphs. It supports input for molecular files in the SMILES, SLN or SDfile formats.

#### Comparison of tools

To give an overview of the performance of the various subgraph mining algorithms we evaluated each on a protein structure graph dataset derived from all RCSB PDB non-redundant structures, similar to those described in “Application in bioinformatics” section. The graph statistics are summarized in Table 2. We compared the performance for support thresholds of 50, 30, 20 and 10%. Tables 3 and 4 show CPU time in seconds and memory consumption in MB, respectively. There is no information about memory consumption for AcGM and FSG, since they are binary executables. These values are only indicative as they result from a single run on a single computer.

**Table 2:** Characteristics of the dataset used to evaluate subgraph mining implementations for the multiple graph setting

Number of graphs	12,073
Number of edge labels	1
Number of node labels	21
Average number of edges in a graph	453
Average number of nodes in a graph	197
Max number of edges in a graph	16,199
Max number of nodes in a graph	6642

**Table 3:** Time elapsed in seconds – tools for the multiple graph setting

Algorithm	Support (%)	10	20	30	50
AcGM		>7200	>7200	>7200	303.7
FSG		>7200	2883.8	805.2	46.1
GASTON		685.1	176.8	79.5	21.8
gSpan		2226.7	402.1	152.3	59.3
lFFSM		1639.5	316.7	122.2	30.5
MoFa/MOSS		759.4	144.2	56.5	19.7

**Table 4:** Memory consumption in MB – tools for the multiple graph setting

Algorithm	Support (%)	10	20	30	50
Gaston		1,147	743	679	624
gSpan		996	559	511	493
FFSM		1,513	1,059	895	678
MoFa/MOSS		1,700	1,200	980	549

The ParMol package contains implementations of four subgraph mining algorithms and is convenient in case the user wants to test several algorithms on the same dataset. However, MoFa/MoSS is more user-friendly than the ParMol package, and accepts a wide variety of input formats. Testing our example graph database, AcGM had a running time one order of magnitude slower than its competitors. FSG was the second slowest algorithm with a running time one order of magnitude slower than GASTON, gSpan, FFSM and MoFa/MoSS for a support threshold of 20% and lower. For a lower support threshold (10%) GASTON and MoFa/MoSS had a two-fold faster running time than gSpan and FFSM.

#### Interpretation of the results

The output of the tools for mining subgraphs in multiple graph settings is given in Section 10.2. All methods, except for FSG, find the same patterns for all support thresholds. FSG returns a lower number of frequent subgraphs than the remaining algorithms because it does not count one node subgraphs. As the input concerned a large number of protein molecular structures encoded as graphs, the output graph patterns concern configurations of amino acids that are common within the molecular structure. For example, one of the three-node subgraphs that was found with high frequency (50.88%) was LEU-GLU-ARG. The close proximity of glutamate and arginine in this subgraph are indicative of a salt bridge, a common configuration in protein structures. Another example with high frequency (40.23%) was THR-VAL-PHE, which may be indicative of a *β*-sheet as all three amino acids are known to be preferred in the middle of *β*-sheets and thus would be in close proximity.

### In a single graph

#### Application in bioinformatics

Much of today’s biomedical relationship data is represented as a large single graph or network representation. Mining these single graphs involves identifying those subgraphs, substructures, or network motifs that are common, and specifically those that are more common than a given background distribution would suggest.

**Fig. 11:**
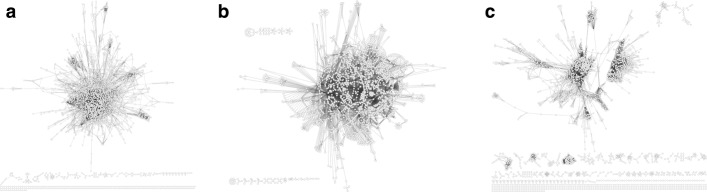
Examples of interaction/association graphs. **a** Escherichia coli interaction network from the bacteriome database [73]. **b** Escherichia coli transcriptional regulatory network from the RegulonDB database [74]. **c** Escherichia coli co-expression network from the Colombos database [75]

**Interaction and association networks** The interactions and associations between the different biomolecules in cells, such as genes, proteins, and metabolites, are most commonly conceptualized as a graph. The biomolecules are featured as the graph nodes. The edges in these graphs can then represent direct interactions, such as protein–protein complexes or kinase–target relationships, but also indirect interactions, such as between genes coding for transcription factor proteins and the gene targets of these transcription factors. For association graphs, the edges are biomolecules that are co-occurring, such as in co-expression networks, or sharing similar functionality. Despite representing different types of relationships, these graphs have very similar properties: they are often very large, featuring several hubs of nodes with a very high degree, and are therefore often described as *hairballs*, as can be seen in Fig. 11. Depending on the relationship type, the edges are either directed for those with a strict regulator-target relationship, or undirected for associations and complexes. Most of the studies in this field are done on graphs that have unlabeled nodes and edges. The objective of subgraph mining interaction and association networks often concerns the identification of those subgraphs, termed graphlets or network motifs, that are present in a much higher (or lower) quantity than in a collection of similarly-sized random networks that function as the background for the relevant statistical test. Given the size of these networks, typically only induced unlabeled subgraphs are examined. These are then generally described as the *building blocks* of the network [46, 47], and have been related to specific biological functions [48]. The identified network motifs can then be used in a number of different contexts, such as as a measure for graph similarity [14], network alignment [49, 50], and network prediction [51]. Several reviews are available that focus on the identification and usage of network motifs in these types of graphs [52–55].

**Other biomedical graphs**

A few other single graph problems are worth mentioning as they demonstrate that subgraph mining in bioinformatics is not limited to molecular data. The first is the mining of brain connectivity maps or neuronal networks [56]. These are graphs that represent the synaptic connections between different neurons. The neurons are thus represented as unlabeled nodes, with directed edges as connections from one neuron to the next. The second involves the mining of food webs that document the relationships between species in an ecosystem [47, 56, 57]. Each node represents a species and directed edges denote predator-prey relationships. In both cases, the goal of the mining is typically the identification of common subgraphs that may represent network building blocks with a specific biological function.

#### Subgraph mining tools available for bioinformatics use

We present a short overview of the algorithms and tools available for mining subgraphs in a single graph. Summarized information can be found in Table 5. It should be noted that we only considered algorithms for which implementations were available. A more detailed overview of the algorithms and tools for motif detection can be found in [58].

**Table 5:** Overview of subgraph mining algorithms and tools for the single graph setting

Algorithm	Interface	Programming language	Website
Mfinder [30]	Command line	C++	[80]
MAVisto [81]	GUI	Java	[82]
FANMOD [83]	GUI	C++	[84]
Kavosh [61]	Command line, Cytoscape plug-in	C++	[85]
NetMODE [62]	Command line	C++	[86]
acc-Motif [63]	Command line	Java	[87]
SSM [24]	Command line	Java	[88]

**Mfinder**

Mfinder is a tool for network motif detection. It contains implementations of algorithms for both exhaustive motif enumeration and motif sampling [46, 56]. Exhaustive motif enumeration looks for all possible subgraphs of a certain size (expressed as a number of edges) that appear more frequently than expected in a random network. Motif sampling for subgraph counting estimates the frequency of subgraphs by sampling within a subset of the whole graph. Random subgraphs are sampled by randomly choosing an edge, and then extending that edge until a subgraph of the required size is obtained. Both algorithms look for subgraphs in a DFS manner and generate candidate subgraphs by adding a new edge in each iteration, i.e. they use edge-based candidate generation. Mfinder is compatible with MDraw, a network visualization tool. Mfinder was the first tool for motif detection and it works with both directed and undirected unlabeled graphs and supports a motif size between 3 and 8. Unfortunately, currently it is no longer under active development.

**MAVisto**

MAVisto is a Java Web Start application that contains an implementation of the frequent pattern finder (FPF) algorithm [59] for motif detection. This algorithm searches for all motifs of a certain size (which can be defined either as a number of nodes or a number of edges) that appear more frequently than in an ensemble of random networks by using one of three different concepts of motif frequency. These frequency concepts differ in the way they allow overlapping matches. FPF builds a pattern tree with the simple one-edge, two-node-graph at its root and expands this by iteratively adding a new edge. It utilizes the downward closure property and spends time only on the promising branches. The MAVisto tool works with both directed and undirected unlabeled graphs, but it is currently no longer under active development.

**FANMOD**

FANMOD is a tool that implements the RAND-ESU algorithm for enumerating and sampling subgraphs [60] as well as the full enumeration algorithm. RAND-ESU was designed to address the bias of the sampling method for subgraph counting implemented in Mfinder, as Mfinder’s sampling method is prone to sampling certain subgraphs more often than others. RAND-ESU fixes this bias and is faster. It enumerates all subgraphs of a certain size, although during the execution it will ignore some of these to achieve an unbiased sampling. FANMOD works with both directed and undirected, labeled and unlabeled graphs and it can detect motifs of up to a size of 8 nodes (min size: 3).

**Kavosh**

The Kavosh algorithm [61] is designed with the goal of finding motifs with less memory and CPU time. It enumerates all subgraphs of a certain size in the graph first by finding all subgraphs that contain a certain node, then removing that node from the graph and repeating the process. Kavosh works with both directed and undirected unlabeled graphs and poses no restriction on the motif size (defined as the number of nodes) to be searched.

**NetMODE**

NetMODE [62] is a software package for motif detection designed specifically as an improvement over Kavosh (upon which it was built) and FANMOD. As opposed to FANMOD, which contains implementations of both sampling and full enumeration algorithms, NetMODE exclusively performs full enumeration. In contrast to Kavosh it searches only for motifs of size 3–6 nodes. Initially much faster than both Kavosh and FANMOD, NetMODE can also be run in parallel. It works with both directed and undirected unlabeled graphs.

**acc-Motif**

The accelerated motif (acc-motif) program [63] implements a motif detection algorithm that counts motifs of size *k* using the set of induced subgraphs of size *k*−2. Here, the size of the motif is equal to the number of nodes. This algorithm is efficient only for smaller motifs (up to size 6). It allows multi-threaded execution and works with both directed and undirected unlabeled graphs.

**Significant Subgraph Miner**

Significant Subgraph Miner (SSM) [24] is an implementation of the algorithm for finding subgraphs associated with a certain set of nodes in a single graph, as per subgroup discovery. The algorithm generates candidate significant subgraphs in a DFS manner, starting with the node of interest as the root node. If significantly more nodes of interest are root nodes for a subgraph compared to the other nodes of the graph (which are not of interest), the subgraph will be deemed as significantly enriched. SSM works with both directed and undirected, labeled and unlabeled graphs.

#### Comparison of tools

To compare these tools we used the protein–protein interaction graph of *Escherichia coli* as present in the Bacteriome database [64] with a confidence cut-off of 0.8. The graph was left unlabeled as only SSM supports labels. The graph statistics are summarized in Table 6 and it is visualized in Fig. 11a. We compared the performance for motif sizes of 3, 4 and 5. The number of randomized networks was 100 for motifs of size 3, 10 for 4-size motifs and 5 for 5-size motifs. All tools were compared in full enumeration mode and a single thread. Table 7 shows CPU time in seconds, while Table 8 shows memory consumption in MB. These are only indicative values as the measurements were made only once on a single computer.

**Table 6:** Characteristics of the dataset used to evaluate subgraph mining implementations for the single graph setting

Number of edge labels	1
Number of node labels	1
Number of edges in a graph	2919
Number of nodes in a graph	821

**Table 7:** Time elapsed in seconds – tools for the single graph setting

Algorithm	Motif size (# random networks)	3 (100)	4 (10)	5 (5)
Mfinder		447	1000	>7200
MAVisto		723	1922	>7200
FANMOD		6	30.6	706.9
Kavosh		4.4	24.1	563.6
NetMODE		0.9	1.6	34.9
acc-Motif		0.3	0.8	20.9
SSM		1.1	5.1	972.2

**Table 8:** Memory consumption in MB – tools for the single graph setting

Algorithm	Motif size (# random networks)	3 (100)	4 (10)	5 (5)
Mfinder		2.7	3.3	-
MAVisto		298	713	-
FANMOD		9.5	9.6	9.9
Kavosh		0.3	0.3	0.3
NetMODE		0.8	0.8	1.8
acc-Motif		26	26	53
SSM		<445	445	520

Mfinder and MAVisto exhibited the longest running times, as was shown in [61]. FANMOD is two orders of magnitude faster than Mfinder and is overall the best tool in terms of user-friendliness. FANMOD supports both a full enumeration and a sampling mode, and works with labeled data as well. As expected Kavosh is slightly faster than FANMOD and consumes less memory. Kavosh also has one big advantage — it does not pose a restriction on the motif size. NetMODE and acc-Motif have similar performances when it comes to speed, they are both one order of magnitude faster than Kavosh with acc-Motif being slightly faster. They both have multi-threading capability. This allows them to be run on graphs that can be too large for FANMOD to handle. However, as opposed to Kavosh they are restricted to motifs of size up to 6 nodes and do not work with labeled graphs. In terms of memory consumption, acc-Motif requires more memory. SSM is quite unique since it does not look for motifs of a specific size (it only has a restriction for maximum edges allowed), but for all subgraphs which are associated with certain nodes. However, it can also enumerate all subgraphs within a graph. In terms of running time, it has roughly a running time of the same order of magnitude as Kavosh. Its memory consumption is one order of magnitude larger than acc-Motif’s.

#### Interpretation of the results

The output of the tools for mining subgraphs in single graph is given in Additional file 2. As the starting graph was a protein-protein interaction network, all methods returned subgraphs that represent configurations of proteins that are frequent or significantly enriched compared to a randomized graph of the same size. Two configurations are common throughout all of the results, independent of the subgraph size that was being investigated. The first are densely connected subgraphs where all nodes have edges that connect to (almost) all other nodes. These are indicative of protein complexes, where many proteins interact with each other. These will co-elute in pull-down experiments and create dense clusters within the graph. For example, MFINDER subgraph 238, featured in the supplementary materials, is a fully connected graph between three nodes. This three-node graph occurred 4683 times in the protein-protein interaction network, but only on average 3429 times in random graphs of a similar size. This graph was therefore enriched with a P-value less than 1×10^−32^ in the protein-protein interaction network. The second type of common enriched configuration is where a single protein interacts with all other proteins, but they (hardly) interact with each other. These subgraphs are indicative of hub proteins, a well known feature of protein-protein interaction networks where a single protein has a large number of interactions. An example is the FANMOD subgraph 4382, which contains a single node that is connected to three other nodes with no other shared edges. However, this subgraph was found to occur less than expected when compared to a random graph, indicating that while hub proteins are present in the graph, they are relatively rare. The differences between the output of the various methods are due to their different search parameters. Mfinder finds fewer motifs than the remaining tools because it calculates motif sizes based on their number of edges instead of their number of nodes. SSM finds more subgraphs because it filters the subgraphs based on a subgroup discovery criterion and to directly compare it to the other approaches the entire graph was added as a single subgroup. The remaining methods found the same number of motifs.

## Conclusions

We have given an overview of the basic principles of subgraph mining and how it can be used for biomedical applications. We made a distinction based on those methods and problems that feature finding frequent subgraphs across multiple graphs and those that find them within a single graph. These approaches are very similar algorithmically. The basic steps thus remain: first graph encoding, then candidate generation, and finally subgraph counting. However they use different criteria to define which graphs are actually interesting.

Despite its broad applicability there are still some enduring issues that prevent subgraph mining in a biomedical setting from reaching its full potential. First, labeled single graph solutions are very rare, yet very relevant for biomedical research [65–69]. The introduction of labels into existing solutions is however non-trivial as it cause a massive explosion of the number of subgraphs that need to be evaluated. In addition, many biological graphs allow multiple labels per node. Another issue when it comes to subgraph mining approaches for single graphs is that many of them struggle with large graphs, especially those with many hubs, i.e. highly connected nodes. As the input graph gets larger, so does the number of potential subgraphs of interests and for a lower frequency threshold, the number of patterns potentially explodes. Recently, there have been solutions that mine for frequent subgraphs in large graphs at low frequency thresholds [70]. There is also a question of the right interestingness measure, i.e. how to define an interesting subgraph from a bioinformatics point of view. The most frequent subgraphs are not necessarily the ones of interest and different alternative measures have been devised from a theoretical computer science perspective [71, 72]. Unfortunately, many of these interestingness measures are not as relevant for biomedical applications. There is thus still a need for novel algorithms which can find biologically relevant subgraphs in a flexible manner.

## Additional files

Additional file 1 More detailed definitions of graphs and subgraphs. (PDF 385 kb)

Additional file 2 Output of tools for both single and multiple graph settings. (PDF 286 kb)

## Abbreviations

AGM: Apriori graph mining
BFS: Breadth-first search
CAM: Canonical adjacency matrix
DFS: Depth-first search
FPF: Frequent pattern finder
MIS: Maximum independent set
