# Supplementary material for: Grasping frequent subgraph mining for bioinformatics applications
Source: BioData Min. 2018 Sep 3;11:20. doi: 10.1186/s13040-018-0181-9 (PMC6122726; doi:10.1186/s13040-018-0181-9)
Supplement: Supplementary file 1 — More detailed definitions of graphs and subgraphs. (PDF 385 kb) [file 13040_2018_181_MOESM1_ESM.pdf]

Additional file 1 – Definitions

Graphs and subgraphs

Graph A graph G is defined as pair $G(V, E)$ consisting of two sets, a set of nodes V and a set of edges $E \subseteq V \times V$ (Figure S1a).

Subgraph We say that graph G_s is a subgraph of graph G if the set of all nodes and the set of all edges of graph G_s are subsets of the set of all nodes and the set of all edges of graph G, respectively. More formally, graph $G_s(V_s, E_s)$ is considered a subgraph of $G(V, E)$, denoted as $G_s \subseteq G$, if the following two conditions are satisfied: (1) $V_s \subseteq V$ and (2) $E_s \subseteq E$. An example is shown in Figure S1e.

Induced subgraph A subgraph G_s is an induced subgraph of a graph G if its set of nodes is a subset of the set of nodes of graph G, $V_s \subseteq V$, and its set of edges, $E_s \subseteq E$, consists of all edges that connect nodes in V_s. In other words, all edges between the selected nodes are preserved. In more formal terms, we say that subgraph G_s is an induced subgraph of a graph G if the following conditions are met:

$$\begin{aligned} V_s \subseteq V, E_s \subseteq E \\ \forall v_i, v_j \in V_s, (v_i, v_j) \in E_s \iff (v_i, v_j) \in E \end{aligned} \tag{1}$$

An example of an induced subgraph is shown in Figure S1f.

Labeled graph If the nodes and edges of a graph have labels, then such a graph is considered a labeled graph. In that case the graph is represented as $G(V, E, L_V, L_E, \lambda, \mu)$, where V is a set of nodes, E is a set of edges; L_V and L_E are sets of all possible node and edge labels, respectively; $\lambda : V \rightarrow L_V$ and $\mu : E \rightarrow L_E$ are functions assigning labels to nodes and edges, respectively. An example of a labeled graph is shown in Figure S1b. Such labels can denote any property of the nodes or edges, for example the amino acid type in protein graphs, or the type of interaction in interaction networks.

Directed and undirected graph A graph is deemed directed if every edge in the graph represents an ordered pair of nodes (illustrated in Figure S1c). If there is no edge orientation in a graph, the graph is deemed undirected (illustrated in Figure S1a). Undirected graphs are common for molecular structures as there is typically no specified direction in the chemical bonds.

Connected and unconnected graph A graph is deemed connected if there is a path along the edges that links each pair of nodes (see Figure S1f for an example); otherwise it is deemed unconnected (Figure S1e). While most complete chemical structures are connected graphs, many protein interaction networks are for example unconnected.

Weighted and unweighted graph If there is a value assigned to each edge in the graph, then the graph is considered to be weighted and the assigned value is called the weight (see Figure S1d). A graph whose edges have no weight is considered unweighted (Figure S1a). These weights can be used to denote the certainty of the edge, as estimated by experimental or computational determination. It can also imply the strength of the interaction (for example affinity in interactions between biomolecules).

Graph isomorphism If there exists a mapping between two graphs such that if two nodes are connected in one graph by an edge, they will be connected in the other graph as well, then such graphs are considered isomorphic. In other words, if two graphs are isomorphic they are considered to be equivalent. The graphs shown in Figure S1a and Figure S1g are isomorphic.

Formally, given two unweighted graphs $G_1(V_1, E_1, L_{V_1}, L_{E_1}, \lambda_1, \mu_1)$ and $G_2(V_2, E_2, L_{V_2}, L_{E_2}, \lambda_2, \mu_2)$, G_1 is isomorphic to G_2, denoted as $G_1 \simeq G_2$, if and only if a bijection $f : V_1 \rightarrow V_2$ exists such that the following conditions are met:

$$\begin{aligned} \forall v \in V_1, \lambda_1(v) = \lambda_2(f(v)) \\ \forall (v_i, v_j) \in E_1 \iff (f(v_i), f(v_j)) \in E_2 \\ \forall (v_i, v_j) \in E_1, \mu_1(v_i, v_j) = \mu_2(f(v_i), f(v_j)) \end{aligned} \tag{2}$$

The bijection f is called an isomorphism between graphs G_1 and G_2. In other words, the isomorphism f preserves both the edge adjacencies and the node and edge labels.

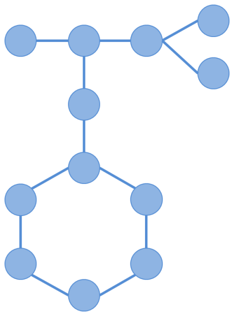

(a) Graph G. Connected, undirected, unlabeled, unweighted.

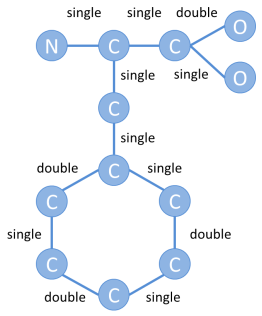

(b) A labeled graph G'.

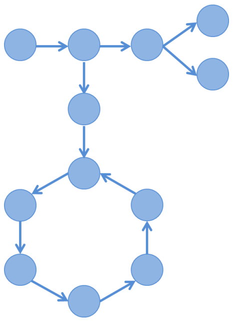

(c) A directed graph G''.

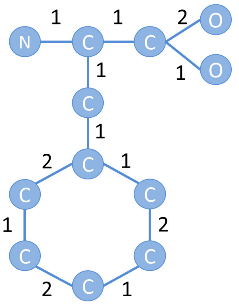

(d) A weighted graph G'''.

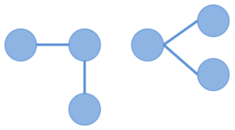

(e) A subgraph of graph G. Non-induced (not all the edges connecting nodes are present).

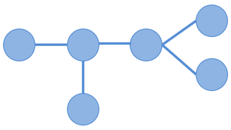

(f) An induced subgraph of graph G.

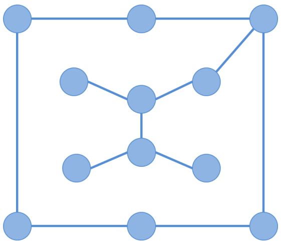

(g) Graph isomorphic to graph G.

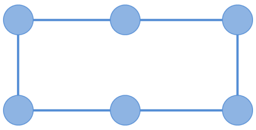

(h) A subgraph isomorphic to graph G.

Figure S1: Graph and subgraphs example.

Subgraph isomorphism If we say that two graphs are isomorphic, it means that we consider those graphs to be equal. If we have two graphs of different sizes, it is clear they cannot be graph isomorphic, i.e. they cannot be equal. However, if the smaller graph is completely within the larger graph, then the graphs are subgraph isomorphic. We say that graphs G_1 and G_2 are subgraph isomorphic, if and only if G_1 contains a subgraph $G_S \subseteq G_1$, such that G_S is isomorphic to G_2, $G_S \simeq G_2$ (Figure S1h).
