# Supplementary material for: Grasping frequent subgraph mining for bioinformatics applications
Source: BioData Min. 2018 Sep 3;11:20. doi: 10.1186/s13040-018-0181-9 (PMC6122726; doi:10.1186/s13040-018-0181-9)
Supplement: Supplementary file 2 — Output of tools for both single and multiple graph settings. (PDF 286 kb) [file 13040_2018_181_MOESM2_ESM.pdf]

Additional file 2 – Output of the tools

1 Multiple graph settings

1.1 AcGM

The output is displayed in command line and not as a file. Subgraphs are given in a DFS code. Returns the same number of frequent subgraphs as ParMol and MoFa/MoSS.

1.2 FSG

Output file contains information about the graph dataset and frequent subgraphs (with absolute support). Returns different number of patterns comparing to other tools, because it does not include one node subgraphs.

- 292 frequent subgraphs for support threshold $\geq$ 50% (the first five frequent subgraphs from file are shown)

```
t # 1-0, 7715
v 0 GLN
v 1 SER
u 0 1 1

t # 1-1, 8562
v 0 GLN
v 1 VAL
u 0 1 1

t # 1-2, 6949
v 0 HIS
v 1 VAL
u 0 1 1

t # 1-3, 6203
v 0 HIS
v 1 SER
u 0 1 1

t # 2-0, 6143
v 0 GLU
v 1 ARG
v 2 LEU
u 0 1 1
u 0 2 1
```

- 2094 frequent subgraphs for support threshold $\geq$ 30% (4 frequent subgraphs from file are shown, one for each subgraph size)

t # 1-0, 7715
v 0 GLN
v 1 SER
u 0 1 1

t # 2-0, 4857
v 0 VAL
v 1 PHE
v 2 THR
u 0 1 1
u 0 2 1

t # 3-0, 3791
v 0 ALA
v 1 LEU
v 2 VAL
u 0 1 1
u 0 2 1
u 1 2 1

t # 4-0, 3742
v 0 LEU
v 1 LEU
v 2 LEU
v 3 ALA
v 4 VAL
u 0 1 1
u 0 2 1
u 1 3 1
u 2 4 1

- 8021 frequent subgraphs for support threshold $\geq 20\%$ (5 frequent subgraphs from file are shown, one for each subgraph size)

```
t # 1-0, 7715
v 0 GLN
v 1 SER
u 0 1 1

t # 2-0, 4857
v 0 VAL
v 1 PHE
v 2 THR
u 0 1 1
u 0 2 1

t # 3-0, 2743
v 0 ILE
v 1 ALA
v 2 VAL
u 0 1 1
u 0 2 1
u 1 2 1

t # 4-0, 2504
v 0 ALA
v 1 LEU
v 2 LEU
v 3 VAL
u 0 1 1
u 0 3 1
u 1 2 1
u 2 3 1

t # 5-0, 2437
v 0 LEU
v 1 LEU
v 2 LEU
v 3 VAL
v 4 ALA
v 5 LEU
u 0 1 1
u 0 2 1
u 1 3 1
u 2 4 1
u 3 5 1
```

1.3 ParMol - GASTON, gSpan, FFSM

Output file contains frequent subgraphs in a form of a node-edge lists, together with the absolute support and the unique identifiers of a graphs containing that subgraph.

For the example dataset following motifs were found:

- 312 frequent subgraphs for support threshold $\geq 50\%$ (the first five frequent subgraphs from file are shown)

t # 24148
v 0 LEU
⇒ [11941.0] [1,2, ... , 12073]

t # 24150
v 0 LEU
v 1 PHE
e 0 1 1
⇒ [9687.0] [1,2, ... , 12073]

t # 24152
v 0 LEU
v 1 PHE
v 2 ARG
e 0 1 1
e 1 2 1
⇒ [6316.0] [1,3, ... , 12072]

t # 24154
v 0 LEU
v 1 PHE
v 2 VAL
e 0 1 1
e 1 2 1
⇒ [6524.0] [1,3, ... , 12064]

t # 24156
v 0 LEU
v 1 PHE
v 2 ILE
e 0 1 1
e 1 2 1
⇒ [6046.0] [1,2, ... , 12041]

- 2114 frequent subgraphs for support threshold $\geq 30\%$ (the first five frequent subgraphs from file are shown)

t # 25752
v 0 LEU
v 1 VAL
v 2 SER
e 0 1 1
e 1 2 1
⇒ [7195.0] [1,2, ... , 12042]

t # 25754
v 0 LEU
v 1 VAL
v 2 SER
v 3 GLY
e 0 1 1
e 1 2 1
e 0 3 1
⇒ [3775.0] [1,3, ... , 11970]

t # 25756
v 0 LEU
v 1 VAL
v 2 SER
v 3 VAL
e 0 1 1
e 1 2 1
e 0 3 1
⇒ [4135.0] [1,2, ... , 11965]

t # 25758
v 0 LEU
v 1 VAL
v 2 SER
v 3 ILE
e 0 1 1
e 1 2 1
e 0 3 1
⇒ [4107.0] [2,3, ... , 11965]

t # 25760
v 0 LEU
v 1 VAL
v 2 SER
v 3 ALA
e 0 1 1
e 1 2 1
e 2 3 1
⇒ [3983.0] [1, 3, ... , 11934]

- 8041 frequent subgraphs for support threshold $\geq 20\%$ (the first five frequent subgraphs from file are shown)

t # 32994
v 0 LEU
v 1 GLU
v 2 SER
e 0 1 1
e 1 2 1
⇒ [5974.0] [2, 3, ... , 12067]

t # 32996
v 0 LEU
v 1 GLU
v 2 SER
v 3 GLY
e 0 1 1
e 1 2 1
⇒ [2537.0] [4, 7, ... , 11960]

t # 32998
v 0 LEU
v 1 GLU
v 2 SER
v 3 VAL
e 0 1 1
e 1 2 1
e 0 3 1
⇒ [3078.0] [7, 8, ... , 11923]

t # 33000
v 0 LEU
v 1 GLU
v 2 SER
v 3 ILE
e 0 1 1
e 1 2 1
e 0 3 1
⇒ [2806.0] [3, 7 ... , 12042]

t # 33002
v 0 LEU
v 1 GLU
v 2 SER
v 3 ALA
e 0 1 1
e 1 2 1
e 2 3 1
⇒ [2658.0] [4, 7, ... , 11828]

- 77,429 frequent subgraphs for support threshold $\geq 10\%$ (the first five frequent subgraphs from file are shown)

t # 163578
v 0 GLY
v 1 THR
v 2 GLN
e 0 1 1
e 0 2 1
⇒ [3215.0] [4, 7, ... , 12012]

t # 163580
v 0 GLY
v 1 THR
v 2 GLN
v 3 LEU
e 0 1 1
e 0 2 1
e 0 3 1
⇒ [1602.0] [4, 8, ... , 11802]

t # 163582
v 0 GLY
v 1 THR
v 2 GLN
v 3 ALA
e 0 1 1
e 0 2 1
e 0 3 1
⇒ [1405.0] [7, 8, ... , 11930]

t # 163584
v 0 GLY
v 1 THR
v 2 GLN
v 3 GLY
e 0 1 1
e 0 2 1
e 0 3 1
⇒ [1240.0] [7,8, ... , 11767]

t # 163586
v 0 GLY
v 1 THR
v 2 GLN
v 3 VAL
e 0 1 1
e 0 2 1
e 0 3 1
⇒ [1325.0] [4, 7, ... , 12012]

1.4 MoFa/MoSS

Output file contains frequent subgraphs in a form of a node-edge lists, together with the information on support value and the unique identifiers of a graphs containing that subgraph. MoFa/MoSS finds the same number of subgraphs as FSG and ParMol.

2 Single graph settings

2.1 Mfinder

Output file includes summary of the input and the results (found motifs and their scores). Found motifs are given in a form of an adjacency matrix.

For the example dataset following motifs were found:

- 1 motif of size 3

  NREAL - Appearances in the real network
  NRAND STATS - Random networks: mean +- SD
  UNIQ VAL - Uniqueness
  CREAL [MILI] - Concentration x 10^{-3}

MOTIF ID	NREAL	NRAND STATS	NREAL ZSCORE	NREAL PVAL	UNIQ VAL	CREAL [MILI]
238	4683	3429.4+-95.6	13.11	0.000	61	58.57

$$\begin{bmatrix} 0 & 1 & 1 \\ 1 & 0 & 1 \\ 1 & 1 & 0 \end{bmatrix}_{ID:238}$$

- 3 motifs of size 4

MOTIF ID	NREAL	NRAND STATS	NREAL ZSCORE	NREAL PVAL	UNIQ VAL	CREAL [MILI]
13260	62824	28882.9+-838.8	40.46	0.000	33	23.33
13278	57155	31668.2+-2300.9	11.08	0.000	28	21.22
31710	6015	2309.7+-374.8	9.89	0.000	21	2.23

$$\begin{bmatrix} 0 & 0 & 1 & 1 \\ 0 & 0 & 1 & 1 \\ 1 & 1 & 0 & 0 \\ 1 & 1 & 0 & 0 \end{bmatrix}_{ID:13260}, \begin{bmatrix} 0 & 1 & 1 & 1 \\ 1 & 0 & 1 & 1 \\ 1 & 1 & 0 & 0 \\ 1 & 1 & 0 & 0 \end{bmatrix}_{ID:13278}, \begin{bmatrix} 0 & 1 & 1 & 1 \\ 1 & 0 & 1 & 1 \\ 1 & 1 & 0 & 1 \\ 1 & 1 & 1 & 0 \end{bmatrix}_{ID:31710}$$

- motifs of size 5 - process killed, took over 2h

2.2 MAVisto

Output motifs shown in GUI. For the example dataset following motifs were found:

- 2 motifs of size 3

motif ID	f1	f2	f3	p-value	z-score	mean	std
FS3	89320	837	71	1	0	89,320	0
HNL	4683	385	62	0	12.611	3,408.34	101.075

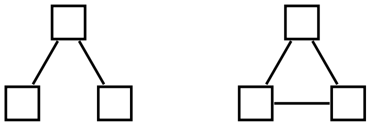

(a) FS3 (b) HNL

- 6 motifs of size 4

motif ID	f1	f2	f3	p-value	z-score	mean	std
PYGYL	2276583	517	43	1	-2.664	2,373,702	34.456,921
PYM13	594137	281	37	0	3.277	504,835.9	27,253.212
QWTU9	138024	306	31	0	20.497	68,252.6	3,404.029
QWTWL	93245	166	29	0	10.004	46,670	4,655,797
TV55L	6015	93	26	0	9.813	2,389.1	369.492
PY6A3	168192	487	47	1	0	1,681,982	0

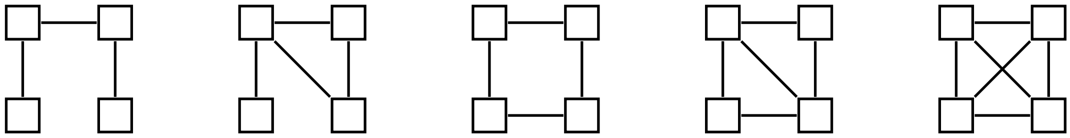

(a) PYGYL (b) PYM13 (c) QWTU9 (d) QWTWL (e) TV55L

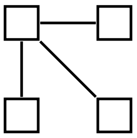

(f) PY6A3

- motifs of size 5 - process killed, took over 2h

2.3 FANMOD

Output file includes summary of the input graph and the results (motifs and their scores). Found motifs are given in a form of an adjacency matrix.

For the example dataset following motifs were found:

- 2 motifs of size 3

ID	Frequency [Original]	Mean-Freq [Random]	Standard-Dev [Random]	Z-Score	p-Value
78	94.143%	99.579%	0.0026476	-20.533	1
238	5.8571%	0.42086%	0.0026476	20.533	0

$$\begin{bmatrix} 0 & 0 & 1 \\ 0 & 0 & 1 \\ 1 & 1 & 0 \end{bmatrix}, \begin{bmatrix} 0 & 1 & 1 \\ 1 & 0 & 1 \\ 1 & 1 & 0 \end{bmatrix}$$
$ID : 78$ $ID : 238$

- 6 motifs of size 4

ID	Frequency [Original]	Mean-Freq [Random]	Standard-Dev [Random]	Z-Score	p-Value
4382	46.425%	50.162%	0.0094988	-3.9347	1
8598	38.005%	44.676%	0.0044247	-15.078	1
4958	10.892%	3.0981%	0.015158	5.1418	0
27030	2.3328%	1.83%	0.0026156	1.9224	0
13278	2.1223%	0.22927%	0.0010909	17.353	0
31710	0.22335%	0.0042406%	5.4663e-005	40.083	0

$$\begin{bmatrix} 0 & 0 & 0 & 1 \\ 0 & 0 & 0 & 1 \\ 0 & 0 & 0 & 1 \\ 1 & 1 & 1 & 0 \end{bmatrix}, \begin{bmatrix} 0 & 0 & 1 & 0 \\ 0 & 0 & 0 & 1 \\ 1 & 0 & 0 & 1 \\ 0 & 1 & 1 & 0 \end{bmatrix}, \begin{bmatrix} 0 & 0 & 0 & 1 \\ 0 & 0 & 1 & 1 \\ 0 & 1 & 0 & 1 \\ 1 & 1 & 1 & 0 \end{bmatrix}, \begin{bmatrix} 0 & 1 & 1 & 0 \\ 1 & 0 & 0 & 1 \\ 1 & 0 & 0 & 1 \\ 0 & 1 & 1 & 0 \end{bmatrix}, \begin{bmatrix} 0 & 0 & 1 & 1 \\ 0 & 0 & 1 & 1 \\ 1 & 1 & 0 & 1 \\ 1 & 1 & 1 & 0 \end{bmatrix}, \begin{bmatrix} 0 & 1 & 1 & 1 \\ 1 & 0 & 1 & 1 \\ 1 & 1 & 0 & 1 \\ 1 & 1 & 1 & 0 \end{bmatrix}$$
$ID : 4382$ $ID : 8598$ $ID : 4958$ $ID : 27030$ $ID : 13278$ $ID : 31710$

- 21 motifs of size 5

ID	Frequency [Original]	Mean-Freq [Random]	Standard-Dev [Random]	Z-Score	p-Value
1083578	37.122%	44.875%	0.0060603	-12.793	1
1082430	22.879%	22.943%	0.0053436	-0.11967	0.6
2133644	13.68%	18.985%	0.0051375	-10.325	1
1084606	5.6947%	2.4627%	0.0068993	4.6847	0
2133678	5.3019%	2.8949%	0.0085506	2.815	0
1150364	5.2433%	5.199%	0.0071103	0.062238	0.6
8948910	2.9151%	1.0963%	0.0033201	5.4781	0
1150398	2.4901%	0.50801%	0.0016326	12.14	0
1256886	1.6522%	0.35228%	0.0010425	12.47	0
3248028	0.53726%	0.22%	0.00051332	6.1806	0
11046574	0.51536%	0.1961%	0.00043116	7.4046	0
5361086	0.40345%	0.039124%	0.00016855	21.615	0
1289662	0.37564%	0.021801%	0.00017425	20.306	0
3248062	0.31537%	0.019644%	5.1987e-005	56.884	0
13225390	0.2381%	0.03316%	6.078e-005	33.716	0
3387326	0.19267%	0.0049404%	3.9655e-005	47.34	0
9997502	0.16604%	0.030197%	0.00013896	9.7755	0
13190438	0.11652%	0.11475%	0.00032081	0.055302	0.4
14273982	0.091156%	0.0035725%	1.0579e-005	82.792	0
7598014	0.063719%	0.00050698%	4.8538e-006	130.23	0
16510910	0.0058196%	1.4158e-005%	2.2466e-007	258.41	0

$$\begin{bmatrix} 0 & 0 & 0 & 0 & 1 \\ 0 & 0 & 0 & 0 & 1 \\ 0 & 0 & 0 & 1 & 0 \\ 0 & 0 & 1 & 0 & 1 \\ 1 & 1 & 0 & 1 & 0 \end{bmatrix}, \begin{bmatrix} 0 & 0 & 0 & 0 & 1 \\ 0 & 0 & 0 & 0 & 1 \\ 0 & 0 & 0 & 0 & 1 \\ 0 & 0 & 0 & 0 & 1 \\ 1 & 1 & 1 & 1 & 0 \end{bmatrix}, \begin{bmatrix} 0 & 0 & 0 & 1 & 0 \\ 0 & 0 & 0 & 0 & 1 \\ 0 & 0 & 0 & 1 & 1 \\ 1 & 0 & 1 & 0 & 0 \\ 0 & 1 & 1 & 0 & 0 \end{bmatrix}, \begin{bmatrix} 0 & 0 & 0 & 0 & 1 \\ 0 & 0 & 0 & 0 & 1 \\ 0 & 0 & 0 & 1 & 1 \\ 0 & 0 & 1 & 0 & 1 \\ 1 & 1 & 1 & 1 & 0 \end{bmatrix} \begin{bmatrix} 0 & 0 & 0 & 1 & 0 \\ 0 & 0 & 0 & 0 & 1 \\ 0 & 0 & 0 & 1 & 1 \\ 1 & 0 & 1 & 0 & 1 \\ 0 & 1 & 1 & 1 & 0 \end{bmatrix}$$
$ID : 1083578$ $ID : 1082430$ $ID : 2133644$ $ID : 1084606$ $ID : 2133678$

$$\begin{bmatrix}0&0&0&0&1\\0&0&0&1&1\\0&0&0&1&1\\0&1&1&0&0\\1&1&1&0&0\end{bmatrix}, \begin{bmatrix}0&1&0&0&0\\1&0&0&0&1\\0&0&0&1&1\\0&0&1&0&1\\0&1&1&1&0\end{bmatrix}, \begin{bmatrix}0&0&0&0&1\\0&0&0&1&1\\0&0&0&1&1\\0&1&1&0&1\\1&1&1&1&0\end{bmatrix}, \begin{bmatrix}0&0&0&0&1\\0&0&1&1&0\\0&1&0&1&1\\0&1&1&0&1\\1&0&1&1&0\end{bmatrix}, \begin{bmatrix}0&0&0&1&1\\0&0&0&1&1\\0&0&0&1&1\\1&1&1&0&0\\1&1&1&0&0\end{bmatrix},$$

$ID : 1150364$ $ID : 8948910$ $ID : 1150398$ $ID : 1256886$ $ID : 3248028$

$$\begin{bmatrix}0&1&0&1&0\\1&0&0&0&1\\0&0&0&1&1\\1&0&1&0&1\\0&1&1&1&0\end{bmatrix}, \begin{bmatrix}0&0&1&0&1\\0&0&0&1&1\\1&0&0&1&1\\0&1&1&0&1\\1&1&1&1&0\end{bmatrix}, \begin{bmatrix}0&0&0&0&1\\0&0&1&1&1\\0&1&0&1&1\\0&1&1&0&1\\1&1&1&1&0\end{bmatrix}, \begin{bmatrix}0&0&0&1&1\\0&0&0&1&1\\0&0&0&1&1\\1&1&1&0&1\\1&1&1&1&0\end{bmatrix}, \begin{bmatrix}0&1&1&0&0\\1&0&0&1&1\\1&0&0&1&1\\0&1&1&0&1\\0&1&1&1&0\end{bmatrix}$$

$ID : 11046574$ $ID : 5361086$ $ID : 1289662$ $ID : 3248062$ $ID : 13225390$

$$\begin{bmatrix}0&0&0&1&1\\0&0&1&1&1\\0&1&0&1&1\\1&1&1&0&1\\1&1&1&1&0\end{bmatrix}, \begin{bmatrix}0&1&0&0&1\\1&0&0&0&1\\0&0&0&1&1\\0&0&1&0&1\\1&1&1&1&0\end{bmatrix}, \begin{bmatrix}0&1&1&0&0\\1&0&0&1&0\\1&0&0&0&1\\0&1&0&0&1\\0&0&1&1&0\end{bmatrix}, \begin{bmatrix}0&1&1&0&1\\1&0&0&1&1\\1&0&0&1&1\\0&1&1&0&1\\1&1&1&1&0\end{bmatrix}, \begin{bmatrix}0&0&1&1&1\\0&0&1&1&1\\1&1&0&1&1\\1&1&1&0&1\\1&1&1&1&0\end{bmatrix},$$

$ID : 3387326$ $ID : 9997502$ $ID : 13190438$ $ID : 14273982$ $ID : 7598014$

$$\begin{bmatrix}0&1&1&1&1\\1&0&1&1&1\\1&1&0&1&1\\1&1&1&0&1\\1&1&1&1&0\end{bmatrix},$$

$ID : 16510910$

2.4 Kavosh

Output includes two files: one with the adjacency matrices of the found motifs and the one with the motifs' scores. For the example dataset Kavosh found the same number of motifs as FANMOD.

2.5 NetMODE

Output includes two files: the file with the adjacency matrices of the found motifs and the file with the motifs' scores. NetMODE found the same number of motifs as FANMOD and Kavosh.

2.6 acc-Motif

Output includes one file with found motifs and their scores, where motifs are given a unique identifier. The package includes folders with motif figures (one folder for each motif size). Based on motif's identifier, user can find motif's figure.

For the example dataset following motifs were found:

- 2 motifs of size 3

Motif Id	Frequency (original)	Mean Frequency (random)	Standard deviation	Z-Score	P-value	Frequency (original) Relative	Mean Frequency (random)	Standard deviation Relative	Z-Score Relative	P-value Relative
111122	3912580	4191246.280	3531.699	-78.904	1.000000	0.951029	0.974748	0.000288	-82.461440	1.000000
222222	201467	108578.240	1177.233	78.904	0.000000	0.048971	0.025252	0.000288	82.461440	0.000000

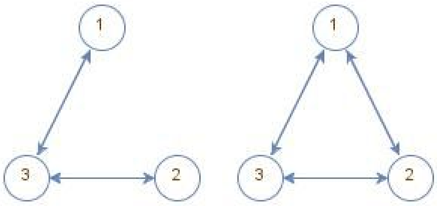

(a) 111122 (b) 222222

- 6 motifs of size 4

Motif Id	Frequency (original)	Mean Frequency (random)	Standard deviation	Z-Score	P-value	Frequency (original) Relative	Mean Frequency (random)	Standard deviation Relative	Z-Score Relative	P-value Relative
274314350350	92202216	67252472.000	637891.357	39.113	0.000000	0.093021	0.065368	0.000640	43.233853	0.000000
293293293293	660598	2185179.700	70119.699	-21.743	1.000000	0.000666	0.002124	0.000068	-21.567579	1.000000
293293726726	8924575	5394683.600	87277.975	40.444	0.000000	0.009004	0.005244	0.000086	43.568092	0.000000
453453843843	96686805	126411958.800	221294.060	-134.324	1.000000	0.097546	0.122870	0.000178	-142.193648	1.000000
494618618618	791515173	827323431.400	843354.439	-42.459	1.000000	0.798545	0.804143	0.000599	-9.350922	1.000000
677677677677	1207565	257882.100	9285.774	102.273	0.000000	0.001218	0.000251	0.000009	106.432574	0.000000

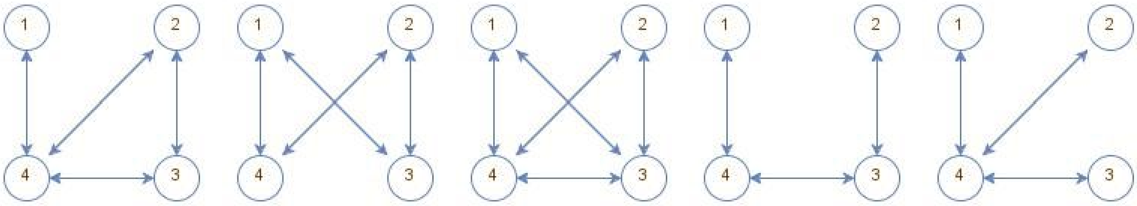

(a) 274314350350 (b) 293293293293 (c) 293293726726 (d) 453453843843 (e) 494618618618

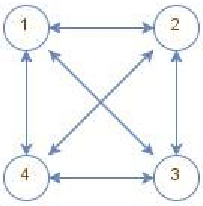

(f) 677677677677

- 21 motifs of size 5

Motif Id	Frequency (original)	Mean Frequency (random)	Standard deviation	Z-Score	P-value	Frequency (original) Relative	Mean Frequency (random)	Standard deviation Relative	Z-Score Relative	P-value Relative
6464838386	183285330	832856207.000	472451351.470	-1.375	1.000000	0.000771	0.000447	0.000048	6.808137	0.000000
6161658658758	7877660	134109126.200	98236526.051	-1.285	1.000000	0.000033	0.000064	0.000009	-3.396690	1.000000
6589811907907	965457913	7636747534.200	5003845267.105	-1.333	1.000000	0.004062	0.003860	0.000075	2.697158	0.000000
8282407476476	1544541374	26138540012.600	19045037307.560	-1.291	1.000000	0.006499	0.012537	0.001627	-3.710437	1.000000
9797657680680	5907880605	46816794114.000	30907424913.809	-1.324	1.000000	0.024858	0.023594	0.000596	2.120207	0.000000
65334351740740	22702918977	234585033634.800	160981477042.043	-1.316	1.000000	0.095525	0.115969	0.007535	-2.713148	1.000000
92197430430875	255030844	7488904414.200	5678930160.073	-1.274	1.000000	0.001073	0.003507	0.000647	-3.759769	1.000000
92616656922922	4819849756	32022299383.000	19995234132.308	-1.360	1.000000	0.020280	0.016523	0.000419	8.973747	0.000000
95325430905905	651173849	4572189242.600	2935369092.165	-1.336	1.000000	0.002740	0.002333	0.000023	17.560919	0.000000
219219270270777	48768638	844458317.800	616534594.233	-1.291	1.000000	0.000205	0.000404	0.000054	-3.693151	1.000000
232608608746746	24254650395	166261728843.400	103347651235.362	-1.374	1.000000	0.102054	0.085876	0.002200	7.353685	0.000000
252252252719782	655099023	2799248101.400	1554157031.278	-1.380	1.000000	0.002756	0.001516	0.000187	6.633413	0.000000
272272500913913	255309590	1676479947.800	1055065622.999	-1.347	1.000000	0.001074	0.000863	0.000018	11.745278	0.000000
352352352352394	6311373	56113906.800	37632958.829	-1.323	1.000000	0.000027	0.000028	0.000001	-1.202477	0.800000
357357357483483	354792652	2325751828.200	1442879109.755	-1.366	1.000000	0.001493	0.001204	0.000037	7.721990	0.000000
473473473939939	2545304	220217382.400	175773961.016	-1.238	1.000000	0.000011	0.000099	0.000028	-3.202203	1.000000
519519519519588	174743900046	1423017694328.200	895075644262.147	-1.395	1.000000	0.735254	0.730212	0.007734	0.651865	0.400000
580580580580580	5933122	19193264.200	9339602.183	-1.420	1.000000	0.000025	0.000011	0.000002	6.189638	0.000000
599654654654654	262651136	1487152803.600	894465991.708	-1.369	1.000000	0.001105	0.000780	0.000045	7.310800	0.000000
763763763879879	30019526	119546698.200	64593800.532	-1.386	1.000000	0.000126	0.000065	0.000009	6.564644	0.000000
948948948948948	6779171	228615021.800	175398287.254	-1.265	1.000000	0.000029	0.000107	0.000020	-3.846679	1.000000

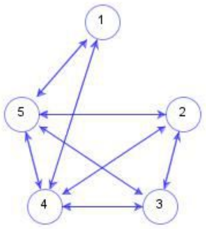

(a) 6464838386

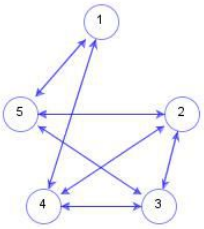

(b) 6161658658758

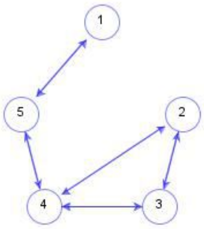

(c) 6589811907907

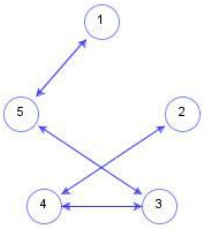

(d) 8282407476476

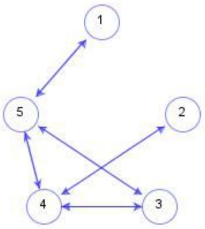

(e) 9797657680680

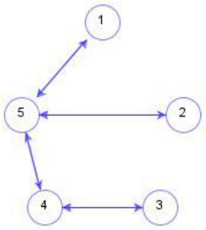

(f) 65334351740740

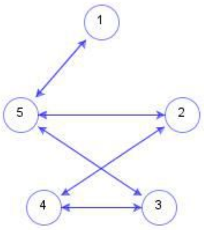

(g) 92197430430875

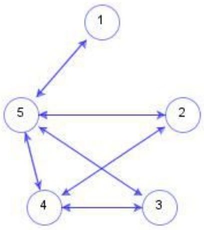

(h) 92616656922922

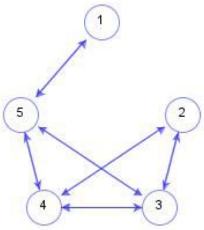

(i) 95325430905905

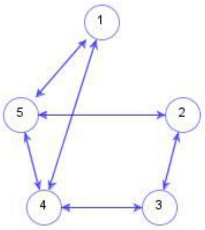

(j) 219219270270777

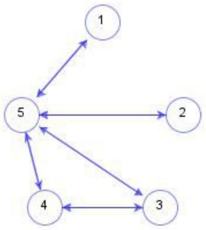

(k) 232608608746746

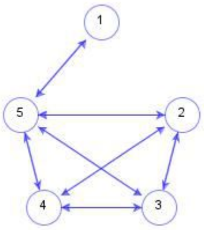

(l) 252252252719782

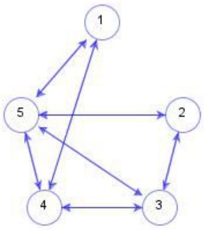

(m) 272272500913913

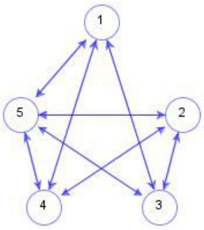

(n) 352352352352394

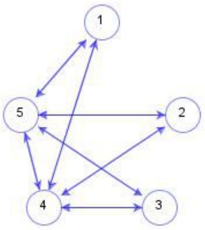

(o) 357357357483483

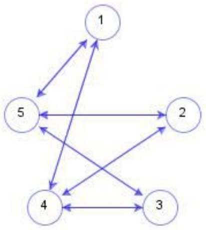

(p) 473473473939939

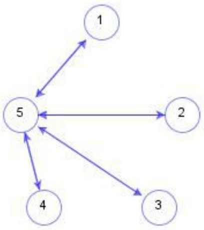

(q) 519519519519588

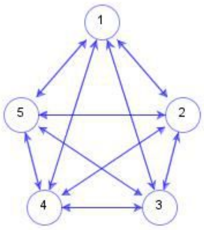

(r) 580580580580580

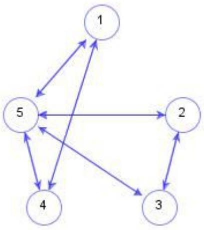

(s) 599654654654654

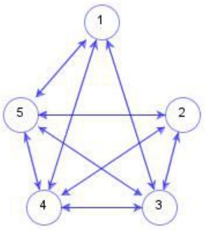

(t) 763763763879879

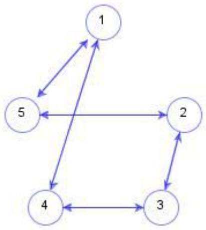

(u) 948948948948948

2.7 Significant Subgraph Miner

Output file includes summary of the input graph and the results (found subgraph and their scores). Found motifs are given in a form of an edge list.

For the example dataset following motifs were found:

- 4 subgraphs with number of nodes less or equal than 3

1 – 2 821 821 0.9999999518659035
1 – 2, 1 – 3 533 533 0.9999999518640845
1 – 2, 2 – 3, 1 – 3 361 361 0.9999999518659035
1 – 2, 2 – 3 743 743 0.9999999518659035

- 17 subgraphs with number of nodes less or equal than 4

1 – 2 821 821 0.9999999518659035
1 – 2, 1 – 3 533 533 0.9999999518640845
1 – 2, 1 – 3, 1 – 4 383 383 0.9999999518659035
1 – 2, 2 – 3, 1 – 3, 1 – 4 320 320 0.9999999518659035
1 – 2, 2 – 3, 2 – 4, 1 – 3, 1 – 4 249 249 0.9999999518640845
1 – 2, 2 – 3, 2 – 4, 1 – 3, 3 – 4, 1 – 4 189 189 0.9999999518640845
1 – 2, 2 – 4, 2 – 3, 1 – 3, 1 – 4 249 249 0.9999999518640845
1 – 2, 2 – 3, 1 – 3 361 361 0.9999999518659035
1 – 2, 2 – 3, 2 – 4, 1 – 3 361 361 0.9999999518659035
1 – 2, 2 – 3, 2 – 4, 1 – 3, 3 – 4 326 326 0.9999999518640845
1 – 2, 2 – 4, 2 – 3, 1 – 3 361 361 0.9999999518659035
1 – 2, 2 – 4, 1 – 3 527 527 0.9999999518640845
1 – 2, 2 – 4, 1 – 3, 3 – 4 437 437 0.9999999518640845
1 – 2, 2 – 3 743 743 0.9999999518659035
1 – 2, 2 – 3, 2 – 4 693 693 0.9999999518659035
1 – 2, 2 – 3, 2 – 4, 3 – 4 644 644 0.9999999518640845
1 – 2, 2 – 3, 3 – 4 727 727 0.9999999518659035

- 115 subgraphs with number of nodes less or equal than 5

1 – 2 821 821 0.9999999518659035
1 – 2, 1 – 3 533 533 0.9999999518640845
1 – 2, 1 – 3, 1 – 4 383 383 0.9999999518659035
1 – 2, 1 – 3, 1 – 4, 1 – 5 296 296 0.9999999518640845
1 – 2, 2 – 3, 1 – 3, 1 – 4, 1 – 5 271 271 0.9999999518640845
1 – 2, 2 – 3, 2 – 4, 1 – 3, 1 – 4, 1 – 5 234 234 0.9999999518659035
1 – 2, 2 – 3, 2 – 4, 2 – 5, 1 – 3, 1 – 4, 1 – 5 191 191 0.9999999518659035
1 – 2, 2 – 3, 2 – 4, 2 – 5, 1 – 3, 3 – 4, 1 – 4, 1 – 5 178 178 0.9999999518659035
1 – 2, 2 – 3, 2 – 4, 2 – 5, 1 – 3, 3 – 4, 3 – 5, 1 – 4, 1 – 5 161 161 0.9999999518640845
1 – 2, 2 – 3, 2 – 4, 2 – 5, 1 – 3, 3 – 4, 3 – 5, 1 – 4, 4 – 5, 1 – 5 136 136 0.9999999518640845
1 – 2, 2 – 3, 2 – 4, 2 – 5, 1 – 3, 3 – 5, 3 – 4, 1 – 4, 1 – 5 161 161 0.9999999518640845
1 – 2, 2 – 3, 2 – 4, 1 – 3, 3 – 4, 1 – 4, 1 – 5 184 184 0.9999999518640845
1 – 2, 2 – 4, 2 – 5, 2 – 3, 1 – 3, 3 – 4, 1 – 4, 1 – 5 178 178 0.9999999518659035
1 – 2, 2 – 5, 2 – 3, 2 – 4, 1 – 3, 3 – 4, 1 – 4, 1 – 5 178 178 0.9999999518659035
1 – 2, 2 – 3, 2 – 4, 1 – 3, 3 – 5, 1 – 4, 1 – 5 198 198 0.9999999518640845
1 – 2, 2 – 3, 2 – 4, 1 – 3, 3 – 5, 1 – 4, 4 – 5, 1 – 5 170 170 0.9999999518640845
1 – 2, 2 – 3, 2 – 5, 2 – 4, 1 – 3, 3 – 5, 3 – 4, 1 – 4, 1 – 5 161 161 0.9999999518640845
1 – 2, 2 – 4, 2 – 3, 1 – 3, 1 – 4, 1 – 5 234 234 0.9999999518659035
1 – 2, 2 – 3, 1 – 3, 1 – 4, 4 – 5, 1 – 5 214 214 0.9999999518659035
1 – 2, 2 – 3, 1 – 3, 1 – 4 320 320 0.9999999518659035
1 – 2, 2 – 3, 2 – 4, 1 – 3, 1 – 4 249 249 0.9999999518640845
1 – 2, 2 – 3, 2 – 4, 2 – 5, 1 – 3, 1 – 4 247 247 0.9999999518640845
1 – 2, 2 – 3, 2 – 4, 2 – 5, 1 – 3, 3 – 4, 1 – 4 189 189 0.9999999518640845
1 – 2, 2 – 3, 2 – 4, 2 – 5, 1 – 3, 3 – 4, 3 – 5, 1 – 4 189 189 0.9999999518640845
1 – 2, 2 – 3, 2 – 4, 2 – 5, 1 – 3, 3 – 4, 3 – 5, 1 – 4, 4 – 5 177 177 0.9999999518659035
1 – 2, 2 – 3, 2 – 4, 2 – 5, 1 – 3, 3 – 5, 3 – 4, 1 – 4 189 189 0.9999999518640845
1 – 2, 2 – 3, 2 – 4, 2 – 5, 1 – 3, 3 – 5, 1 – 4 246 246 0.9999999518659035
1 – 2, 2 – 3, 2 – 4, 2 – 5, 1 – 3, 3 – 5, 1 – 4, 4 – 5 217 217 0.9999999518640845
1 – 2, 2 – 3, 2 – 4, 1 – 3, 3 – 4, 1 – 4 189 189 0.9999999518640845
1 – 2, 2 – 4, 2 – 5, 2 – 3, 1 – 3, 3 – 4, 1 – 4 189 189 0.9999999518640845
1 – 2, 2 – 5, 2 – 3, 2 – 4, 1 – 3, 3 – 4, 1 – 4 189 189 0.9999999518640845
1 – 2, 2 – 3, 2 – 4, 1 – 3, 3 – 5, 1 – 4 249 249 0.9999999518640845
1 – 2, 2 – 3, 2 – 4, 1 – 3, 3 – 5, 1 – 4, 4 – 5 234 234 0.9999999518659035
1 – 2, 2 – 3, 2 – 5, 2 – 4, 1 – 3, 3 – 5, 3 – 4, 1 – 4 189 189 0.9999999518640845
1 – 2, 2 – 3, 2 – 5, 1 – 3, 1 – 4 320 320 0.9999999518659035
1 – 2, 2 – 3, 2 – 5, 1 – 3, 3 – 4, 1 – 4 249 249 0.9999999518640845
1 – 2, 2 – 3, 2 – 5, 1 – 3, 3 – 4, 1 – 4, 1 – 5 198 198 0.9999999518640845
1 – 2, 2 – 3, 2 – 5, 1 – 3, 3 – 4, 1 – 4, 4 – 5, 1 – 5 170 170 0.9999999518640845
1 – 2, 2 – 3, 2 – 5, 2 – 4, 1 – 3, 3 – 4, 3 – 5, 1 – 4, 1 – 5 161 161 0.9999999518640845
1 – 2, 2 – 4, 2 – 5, 2 – 3, 1 – 3, 3 – 5, 1 – 4 246 246 0.9999999518659035
1 – 2, 2 – 3, 2 – 5, 1 – 3, 3 – 4, 1 – 4, 4 – 5 234 234 0.9999999518659035
1 – 2, 2 – 4, 2 – 5, 2 – 3, 1 – 3, 3 – 5, 1 – 4, 4 – 5 217 217 0.9999999518640845
1 – 2, 2 – 3, 2 – 5, 1 – 3, 3 – 5, 1 – 4 297 297 0.9999999518659035
1 – 2, 2 – 3, 2 – 5, 1 – 3, 3 – 5, 1 – 4, 4 – 5 260 260 0.9999999518640845
1 – 2, 2 – 3, 2 – 5, 1 – 3, 1 – 4, 4 – 5 305 305 0.9999999518640845
1 – 2, 2 – 4, 2 – 3, 1 – 3, 1 – 4 249 249 0.9999999518640845
1 – 2, 2 – 5, 2 – 3, 1 – 3, 1 – 4 320 320 0.9999999518659035
1 – 2, 2 – 3, 1 – 3, 1 – 4, 4 – 5 318 318 0.9999999518640845
1 – 2, 2 – 5, 2 – 3, 1 – 3, 1 – 4, 4 – 5 305 305 0.9999999518640845
1 – 2, 2 – 5, 1 – 3, 1 – 4 382 382 0.9999999518659035
1 – 2, 2 – 5, 1 – 3, 3 – 4, 1 – 4 318 318 0.9999999518640845
1 – 2, 2 – 5, 1 – 3, 3 – 4, 1 – 4, 1 – 5 214 214 0.9999999518659035
1 – 2, 2 – 4, 2 – 5, 1 – 3, 3 – 5, 1 – 4 305 305 0.9999999518640845
1 – 2, 2 – 4, 2 – 5, 1 – 3, 3 – 4, 3 – 5, 1 – 4 234 234 0.9999999518659035
1 – 2, 2 – 4, 2 – 5, 1 – 3, 3 – 4, 3 – 5, 1 – 4, 1 – 5 170 170 0.9999999518640845
1 – 2, 2 – 5, 2 – 3, 2 – 4, 1 – 3, 3 – 5, 1 – 4, 4 – 5 217 217 0.9999999518640845
1 – 2, 2 – 5, 2 – 4, 1 – 3, 3 – 5, 1 – 4 305 305 0.9999999518640845
1 – 2, 2 – 5, 1 – 3, 3 – 5, 1 – 4 364 364 0.9999999518640845
1 – 2, 2 – 5, 1 – 3, 3 – 5, 1 – 4, 4 – 5 289 289 0.9999999518659035

1 − 2, 2 − 3, 1 − 3 361 361 0.9999999518659035
1 − 2, 2 − 3, 2 − 4, 1 − 3 361 361 0.9999999518659035
1 − 2, 2 − 3, 2 − 4, 2 − 5, 1 − 3 360 360 0.9999999518659035
1 − 2, 2 − 3, 2 − 4, 2 − 5, 1 − 3, 3 − 4 326 326 0.9999999518640845
1 − 2, 2 − 3, 2 − 4, 2 − 5, 1 − 3, 3 − 4, 3 − 5 300 300 0.9999999518659035
1 − 2, 2 − 3, 2 − 4, 2 − 5, 1 − 3, 3 − 4, 3 − 5, 4 − 5 285 285 0.9999999518659035
1 − 2, 2 − 3, 2 − 4, 2 − 5, 1 − 3, 3 − 4, 4 − 5 320 320 0.9999999518659035
1 − 2, 2 − 3, 2 − 4, 2 − 5, 1 − 3, 4 − 5 355 355 0.9999999518659035
1 − 2, 2 − 3, 2 − 4, 1 − 3, 3 − 4 326 326 0.9999999518640845
1 − 2, 2 − 4, 2 − 5, 2 − 3, 1 − 3, 3 − 4 326 326 0.9999999518640845
1 − 2, 2 − 3, 2 − 4, 1 − 3, 3 − 4, 4 − 5 326 326 0.9999999518640845
1 − 2, 2 − 4, 2 − 5, 2 − 3, 1 − 3, 3 − 4, 4 − 5 320 320 0.9999999518659035
1 − 2, 2 − 3, 2 − 4, 1 − 3, 3 − 5 349 349 0.9999999518659035
1 − 2, 2 − 3, 2 − 4, 1 − 3, 3 − 5, 4 − 5 342 342 0.9999999518640845
1 − 2, 2 − 3, 2 − 4, 1 − 3, 4 − 5 361 361 0.9999999518659035
1 − 2, 2 − 4, 2 − 3, 1 − 3 361 361 0.9999999518659035
1 − 2, 2 − 4, 1 − 3 527 527 0.9999999518640845
1 − 2, 2 − 4, 2 − 5, 1 − 3 516 516 0.9999999518640845
1 − 2, 2 − 4, 2 − 5, 1 − 3, 3 − 4 437 437 0.9999999518640845
1 − 2, 2 − 4, 2 − 5, 1 − 3, 3 − 4, 3 − 5 401 401 0.9999999518640845
1 − 2, 2 − 4, 2 − 5, 1 − 3, 3 − 4, 3 − 5, 4 − 5 372 372 0.9999999518640845
1 − 2, 2 − 4, 2 − 5, 1 − 3, 3 − 4, 4 − 5 427 427 0.9999999518640845
1 − 2, 2 − 5, 2 − 3, 2 − 4, 1 − 3, 3 − 5, 1 − 4 246 246 0.9999999518659035
1 − 2, 2 − 4, 2 − 5, 1 − 3, 4 − 5 504 504 0.9999999518640845
1 − 2, 2 − 4, 1 − 3, 3 − 4 437 437 0.9999999518640845
1 − 2, 2 − 4, 1 − 3, 3 − 4, 4 − 5 437 437 0.9999999518640845
1 − 2, 2 − 5, 2 − 3, 2 − 4, 1 − 3, 1 − 4 247 247 0.9999999518640845
1 − 2, 2 − 4, 1 − 3, 3 − 5 492 492 0.9999999518659035
1 − 2, 2 − 4, 1 − 3, 3 − 5, 1 − 4 318 318 0.9999999518640845
1 − 2, 2 − 4, 1 − 3, 3 − 5, 1 − 4, 1 − 5 214 214 0.9999999518659035
1 − 2, 2 − 4, 1 − 3, 3 − 5, 4 − 5 468 468 0.9999999518640845
1 − 2, 2 − 4, 1 − 3, 4 − 5 525 525 0.9999999518640845
1 − 2, 2 − 3 743 743 0.9999999518659035
1 − 2, 2 − 3, 2 − 4 693 693 0.9999999518659035
1 − 2, 2 − 3, 2 − 4, 2 − 5 666 666 0.9999999518640845
1 − 2, 2 − 3, 2 − 4, 2 − 5, 3 − 4 637 637 0.9999999518640845
1 − 2, 2 − 3, 2 − 4, 2 − 5, 3 − 4, 3 − 5 608 608 0.9999999518640845
1 − 2, 2 − 3, 2 − 4, 2 − 5, 3 − 4, 3 − 5, 4 − 5 582 582 0.9999999518640845
1 − 2, 2 − 3, 2 − 4, 2 − 5, 1 − 3, 3 − 5, 3 − 4, 4 − 5 285 285 0.9999999518659035
1 − 2, 2 − 3, 2 − 4, 2 − 5, 3 − 5, 3 − 4 608 608 0.9999999518640845
1 − 2, 2 − 3, 2 − 4, 3 − 4 644 644 0.9999999518640845
1 − 2, 2 − 3, 2 − 4, 3 − 4, 3 − 5 644 644 0.9999999518640845
1 − 2, 2 − 3, 2 − 4, 3 − 4, 3 − 5, 4 − 5 627 627 0.9999999518659035
1 − 2, 2 − 5, 2 − 3, 2 − 4, 1 − 3, 3 − 4, 4 − 5 320 320 0.9999999518659035
1 − 2, 2 − 3, 2 − 4, 3 − 5, 3 − 4 644 644 0.9999999518640845
1 − 2, 2 − 3, 2 − 4, 3 − 5 689 689 0.9999999518659035
1 − 2, 2 − 3, 2 − 4, 3 − 5, 4 − 5 663 663 0.9999999518659035
1 − 2, 2 − 3, 3 − 4 727 727 0.999999518659035
1 − 2, 2 − 3, 3 − 4, 3 − 5 715 715 0.9999999518640845
1 − 2, 2 − 4, 2 − 5, 2 − 3, 1 − 3 360 360 0.9999999518659035
1 − 2, 2 − 3, 3 − 4, 3 − 5, 4 − 5 708 708 0.9999999518640845
1 − 2, 2 − 4, 2 − 5, 2 − 3, 1 − 3, 4 − 5 355 355 0.9999999518659035
1 − 2, 2 − 5, 2 − 4, 1 − 3, 3 − 4, 4 − 5 427 427 0.9999999518640845
1 − 2, 2 − 3, 3 − 4, 4 − 5 725 725 0.9999999518640845
1 − 2, 2 − 4, 2 − 3, 1 − 3, 4 − 5 361 361 0.9999999518659035
1 − 2, 2 − 5, 2 − 4, 1 − 3, 3 − 4 437 437 0.9999999518640845
